# A novel enhanced competition of tribes and cooperation of members algorithm for global optimization

**DOI:** 10.1371/journal.pone.0324944

**Published:** 2025-06-02

**Authors:** Yu Liu, Maosheng Fu, Chaochuan Jia, Huaiqing Liu, Zongling Wu, Wei Peng, Zhengyu Liu

**Affiliations:** School of Electronics and Information Engineering, West Anhui University, Lu’an, China; Indiana University School of Medicine, UNITED STATES OF AMERICA

## Abstract

The competition of tribes and cooperation of members algorithm (CTCM) is a novel swarm intelligence algorithm, which increases the diversity of the population to a certain extent through tribal competition and member cooperation mechanisms. However, when dealing with certain complex optimization problems, the algorithm may converge to a local optimal solution prematurely, thereby failing to reach the global optimal solution. To enhance the algorithm’s global optimization capabilities and stability, an enhanced CTCM (CTCMKT) is proposed, which integrates a joint strategy of Kent chaotic mapping and *t*- distribution mutation. This integration effectively prevents premature convergence to local optimal solutions, ensuring that the algorithm does not miss the global optimal solution during the optimization process and the algorithm’s stability is significantly enhanced. CEC2021 and 23 benchmark functions are used to test the effectiveness and feasibility of the CTCMKT. By minimizing the fitness value, the CTCMKT is contrasted with other algorithms. Experimental results reveal that the CTCMKT has a superior global optimization ability compared to these algorithms. It can efficiently balance exploration and exploitation to reach the optimal solution. Additionally, the CTCMKT can effectively boost the convergence speed, calculation accuracy, and stability. Engineering application results show that the improved CTCMKT algorithm can solve practical application problems.

## Introduction

Swarm intelligence optimization algorithms, which are inspired by swarm systems in nature and mainly simulate the behavior of biological groups to solve complex optimization problem [1–4]. Swarm intelligence systems operate on a fundamental concept, which attain globally intelligent behavior by following simple local rules. In recent years, optimization algorithms inspired by swarms have been evolving continuously. These algorithms have demonstrated significant potential across a wide range of optimization tasks [5–8].

In the 1990s, the ant colony algorithm was proposed by M. Dorigo et al, which models the behavior of ants as they search for food [9]. This algorithm exhibits excellent performance when dealing with discrete optimization problems, like the traveling salesman problem. In the 2000s, Karaboga et al. introduced the artificial bee colony algorithm, which simulates the foraging behavior of bees, including three roles: employed bees, observation bees and scout bees [10]. The optimal solution is found through division of labor and cooperation. It has been applied and continuously improved in problems such as function optimization.

From the beginning of the 21st century to the present, extensive research on swarm intelligence algorithms have been proposed, for example, the firefly algorithm (FA), whale optimization algorithm (WOA), sparrow search algorithm (SSA), etc [11–14]. These algorithms simulate the flashing and mutual attraction behavior of fireflies, the predation behavior of whales, etc., and have been widely used in many fields, for example, image processing, machine learning, engineering optimization, and bioinformatics [15–19]. Ang et al. studied the drawbacks of particle swarm optimization (PSO) in dealing with constrained optimization problems and proposed a constrained multi-swarm particle swarm optimization algorithm without velocity [20]. It effectively solves constrained optimization problems through various mechanisms and verifies its good performance on benchmark functions through a large number of simulations. Zhang et al. studied an improved PSO (AMPSO) to address the poor performance of PSO in multi-modal and multi-objective optimization problems [21]. By introducing a dynamic neighborhood learning strategy and offspring competition mechanism, and using a variety of functions to verify and compare to other algorithms, the results show that AMPSO is competitive. Tijjani et al. proposed an enhanced PSO (EBPSO), which uses a dimensionality reduction mechanism and a new position update method [22]. After experimental comparison of multiple algorithms, the classification is more accurate on most data sets, effectively solving the feature selection problem, and EBPSO has better performance. Wu et al. proposed the hybrid ant colony algorithm (HACO), which innovatively updates pheromones, introduces adaptive parameters and mutation operations [23]. Verified by Solomon examples and actual cases, HACO outperforms the basic ant colony algorithm and other intelligent algorithms in most cases, effectively reduces vehicle driving distance and cost, and is practical. Zhang et al. proposed the chaotic particle ant colony algorithm (PSCACO), which innovatively transformed multi-objective solutions into single-objective solutions and introduced a chaotic variable optimization ant colony algorithm [24]. Experimental results indicate that the PSCACO algorithm performs more effectively compared to the contrast algorithms in both benchmark function tests and actual cases, and effectively solves multi-objective optimization problems. Wang et al. proposed the BSAS algorithm, which combines the swarm intelligence algorithm with the feedback-based step size update strategy, improving the ability and efficiency of BAS in handling high-dimensional problems [25].

With the aim of improving the performance of the algorithm, researchers began to explore the mixing of different swarm intelligence algorithms or swarm intelligence algorithms with other types of algorithms [26–29]. Hybrid swarm intelligence algorithms are developed based on swarm intelligence algorithms, aiming to combine the advantages of different algorithms and overcome the limitations of a single algorithm to better solve complex optimization problems. Khodayifar et al. proposed the algorithm by combining the advantages of particle swarm optimization (PSODESA) simulated annealing and differential evolution algorithms [30]. By combining the advantages of the two algorithms, the exploration capability is effectively improved and the risk of falling into local optimality is reduced. Deng et al. proposed a hybrid algorithm, introduced the composite adversarial learning strategy, and combined it with PSO to improve the escape ability from local optimality and local search capabilities [31]. Li et al. proposed a hybrid butterfly and Newton-Raphson swarm intelligence algorithm (BOANRBO) based on adversarial learning to solve the problems of local optimality, slow convergence and low precision of the butterfly optimization algorithm [32]. They improved initialization through adversarial learning, introduced adaptive perceptual modal factors and dynamic exploration probability, and combined with the Newton-Raphson optimizer to enhance exploration capabilities. Pashaei et al. proposed a hybrid gene selection method combining differentially expressed gene (DEG) analysis and hiking optimization algorithm (HOA) [33]. First, relevant genes were screened by DEG analysis, and then the binary variants of HOA, BHOA and the improved version BHOA-CM, were used to optimize gene selection.

The basic CTCM algorithm simulates the herd behavior of animals in nature, especially the competitive relationship between tribes and the cooperative relationship between members within a tribe [34]. Even though the CTCM algorithm increases population diversity to a certain level using the tribe competition and member cooperation mechanism, in complex optimization scenarios, it may converge prematurely to a local optimal solution, causing it to miss the global best solution. This is especially likely to happen when the cooperation between members within the tribe is too close or the competition between tribes is not sufficient. The CTCMKT algorithm effectively avoids converging to the local optimal solution in the optimization process and missing the global optimal solution by introducing a joint strategy of Kent chaotic mapping and *t*-distribution mutation. Kent chaotic mapping can uniformly explore all possible states within a specific value range. This implies that it can conduct wide ranging exploration in the search space, thus enhancing the probability of discovering the global optimal solution [35]. The *t*-distribution mutation can adaptively adjust the characteristics of the mutation according to the number of iterations, and effectively balance the algorithm’s exploration and exploitation capabilities [36]. In the early stages of iteration, the focus is on exploration, and new solution spaces are discovered through larger variation steps; as the iteration proceeds, the focus is on development, and the better solutions found are optimized through smaller variation steps.

The subsequent sections of this paper are structured as follows. First, the principles of the CTCM and CTCMKT algorithms will be explained, respectively. Next, the results of all algorithms will be analyzed. Then, the performance of the algorithms in solving practical engineering problems will be discussed. Ultimately, a summary of the research and outlook for future work are presented.

### CTCM

The CTCM is based on the human group competition and member cooperation behavior. In primitive human society, members formed tribes through random cooperation. Each tribe occupied different resources and migrated to obtain more. The tribe was led by a chief, who decided the migration direction and influenced the development of the tribe. Tribe members explored new lands based on experience and the instructions of the chief. Despite close cooperation, members might replace the chief after discovering more fertile land. Competition between tribes was fierce, conflicts were frequent, and the weak would flee in the opposite direction to reduce losses. Based on these characteristics, the CTCM mathematical model was constructed. The CTCM algorithm adopts member cooperation and tribal competition to solve optimization problem.

### CTCMKT

Introducing Kent chaotic map and *t*-distribution mutation into the basic CTCM algorithm, the advantages of the two strategies are combined to effectively avoid the stagnation of CTCM search and slow convergence. CTCMKT can efficiently coordinate global search capabilities and local search capabilities, thereby significantly enhancing calculation accuracy while improving convergence speed, ensuring that the algorithm achieves efficient and accurate iterative optimization in solving complex problems.

### Initialization with Kent chaotic mapping

According to the characteristics of the primitive tribes in searching for resources, the mathematical model for CTCMKT is bulit. Suppose *p* presents the number of humans in primitive society, *n* is the number of tribes, *i* is the number of humans in one tribe, and *d* represents the dimension of the solution space. And *F*_*n*_ represents the fitness value of the entire primitive society, ***v*** denotes the velocity matrix of the entire primitive human society.

Chaotic theory has been increasingly incorporated into swarm intelligence algorithms, leveraging its features such as randomness, ergodicity, and non – repetitiveness. These attributes serve to boost the diversity within the initialized population, thereby enhancing the algorithm’s optimization capabilities. In contrast to random search methods, chaotic theory enables a more comprehensive and in – depth exploration of the search space.

To maximize the utilization of solution – space information by the initial population’s individuals, the Kent mapping from chaotic theory is integrated into the CTCM algorithm for population initialization enhancement. The mathematical model of the Kent mapping can be represented as Eq. (1).


$$\begin{array}{c} \{\;\;\;\;\begin{array}{cc} {}*20c\;\;\;\;\;\;\;\;\;\;\;\;\;\;x_{n,i,d}^{t+1}=\frac{x_{n,i,d}^{t}}{\mu }, & 0<x_{n,i,d}^{t}<\mu \mathrm{\backslash vspace}1mm\\ x_{n,i,d}^{t+1}=\frac{\left(1-x_{n,i,d}^{t}\right)}{1-\mu }, & \mu \leq x_{n,i,d}^{t}<1 \end{array} \end{array}$$ (1)

Where μ is an adjustable parameter in the interval (0, 1). When μ=0.5,the distribution is basically uniform, in this article sets μ=0.5.

### Exploitation

In primitive tribes, the tribe’s management and future planning are the responsibility of the leader. Most members will obey the leader’s arrangements, but individual members also have their own ideas, so the loyalty of individuals will change as time passes. This loyalty will show chaotic behavior, and the increase in the number of tribes will aggravate this chaotic nature. In this case, the sine chaotic mapping is used to characterize the changes in member loyalty. At the same time, it is believed that the amount of tribes *n* will affect the chaotic state. The loyalty of a single member *r*_*t*_ can be expressed as Eq. (2).


$$\begin{array}{c} r_{t}=\left\{ \;\;\;\begin{array}{cc} \;\;\;\;\;\;\;\;\;\;\;U(0,1) & if\;t=k\cdot i\;k\in \mathbb{Z}\\ \sin\left(\frac{\pi }{2}r_{t-1}\right) & else \end{array}\right. \end{array}$$ (2)

The mixed model of loyalty reflecting social behavior is shown in Equation (2), which consists of two parts: random redistribution and chaotic evolution. The random redistribution represents the intermittent major attitude changes, while the chaotic sine map represents the unpredictability of individual members adjusting their loyalty based on previous values. When the number of tribes increases, then the distinctions and relationships between different tribes are enhanced, the chaotic behavior of loyalty is amplified. Increasing interactions and connections may cause the system to be more sensitive to initial conditions because the *r*_*t*_ value may deviate or fluctuate more. Members communicate with the leader to obtain instructions and combine personal experience to provide information for actions. The way to update the speed matrix is similar to the PSO, which is random and reflects the differences in individual thinking and the difference between following instructions and personal ideas, which can be expressed as Eq. (3).


$$\begin{array}{c} v_{n,i,d}^{t+1}=\frac{3}{5}\cdot v_{n,i,d}^{t}+c_{1}r_{1,n,i,d}^{t}(x_{n,i,d}^{best}-x_{n,i,d}^{t}\;+c_{2}r_{2,n,i,d}^{t}(x_{tribe}^{best}-x_{n,i,d}^{t}) \end{array}$$ (3)

Among them, the speed of the *m*th member of the *n*th tribe at the *t + *1 times is represented by $v_{n,i,d}^{t+1}$,and consider the constant 3/5 as the inertia factor. $v_{n,i,d}^{t}$ refers to the velocity at the *t* times. $x_{n,i,d}^{best}$ indicates the position with the optimal fi*t*ness value discovered by the member across the entire period, and $x_{n,i,d}^{t}$ is the position at *t* time. $x_{tribe}^{best}$ represents the position with the best fitness value found by the tribe over the entire duration. *c*_1_ and *c*_2_ refers to the ways in which *t*ribe members follow their own experiences and comply with the chief’s orders, respectively. *r*_1_ and *r*_2_ are the chaotic loyalty of each member.

### Exploration

When tribal conflicts occur, weaker tribes, finding it hard to compete with stronger ones for resources, are often forced into retreat. This retreat is often in a state of chaotic. Some members flee rapidly because of fear, whereas other members retreat more slowly. The unpredictability of these retreats increases as the number of tribes increases. In random conflicts between tribes, weaker tribes will flee, while stronger tribes will not be affected.

$I_{rival}=rand(n)$ represents a random conflict with one among the n tribes. Then the velocity will be updated by Eq. (4) and Eq. (5).


$$\begin{array}{c} R_{n,m,d}^{t}=c_{3}r_{3,n,i,d}^{t}(x_{n,i,d}^{t}-x_{rival}^{best}) \end{array}$$ (4)

$$\begin{array}{c} v_{n,i,d}^{t+1}=\{\;\;\;\;\begin{array}{cc} {}*20cv_{n,i,d}^{t+1}-R_{n,i,d}^{t}, & \begin{array}{cc} {}*20cif & F_{n}^{best}<F_{rival}^{best} \end{array}\\ v_{n,i,d}^{t+1}, & else \end{array} \end{array}$$ (5)

$x_{rival}^{best}$ means the optimal position found by the opponent. $F_{n}^{best}$ represents the optimal fitness of the *n*th tribe, and $F_{rival}^{best}$ is the optimal fitness of the opponent. *c*_3_ represents the tribe escape coefficient, and $r_{3,n,i,d}^{t}$ is a chaotic random factor indicating the retreat speed. At the same time, the positions of the tribe members are updated by Equation (6).


$$\begin{array}{c} x_{n,i,d}^{t+1}=x_{n,i,d}^{t}+v_{n,i,d}^{t+1} \end{array}$$ (6)

The tribe members $x_{n,i,d}^{t+1}$ need to update their positions within the feasible domain [*x*_min_, *x*_max_], where *x*_min_ is the minimum value of the domain and *x*_max_ is the maximum value of the domain. Once this range is surpassed, a mirror bounce effect will happen. The velocity will reverse by Eq. (7), and both its position information and the position of the tribe member will be rectified.


$$\begin{array}{c} v_{n,i,d}^{t+1}=-v_{n,i,d}^{t+1} \end{array}$$ (7)

### *t*-distribution mutation

The *t* – distribution embodies characteristics inherent to both the Cauchy and Gaussian distribution. In the current approach, the degree of freedom parameter of the *t* – distribution is substituted with the number of algorithm iterations. This substitution enables the *t*-distribution to closely approximate the Cauchy distribution during the initial stages of algorithm iteration. In this stage, the global optimization ability of the algorithm is enhanced. As the number of iterations continues to increase, the *t*-distribution approaches the Gaussian distribution. This process improves the search efficiency of the algorithm in the local range, thereby improving the optimization accuracy of the algorithm.

vIn the CTCMKT algorithm, some members are selected with a certain probability to perform *t*-distribution mutation operations. The formula is described as Eq. (8).


$$\begin{array}{c} x_{new}=x_{rival}^{best}+x_{rival}^{best}\cdot t(iter) \end{array}$$ (8)

Where $x_{new}$ is the position of the tribe member after mutation; $x_{rival}^{best}$ is the original position of the tribe member; *iter* is the current iteration number of the CTCMKT; *t*(*iter*) represents the *t*-distribution function wi*t*h the number of iterations as *t*he degree of freedom. The degree of freedom is continuously changed during the iteration process to achieve the effect of adaptively changing the amplitude of mutation. The adaptively adjusted *t* – distribution mutation enhances population diversity. This enables tribe members *t*o potentially break free from local extreme values and locate the global optimal solution, thus boosting the algorithm’s performance. And the pseudo code is shown in Algorithm 1.

**Algorithm 1** The algorithm of CTCMKT

**Input**:

*T*: the maximum iterations

*p*: the number of humans

*n*: the quantity of tribes

*c*_1_: The experience factor

*c*_2_: The obey factor

*c*_3_: The escape factor

Initialize relevant parameters and a population of *p* humans

**Output**: *x*_best_, *F*_best_

**while** iter<*T*

** for** each human **do**

**  **update loyalty factor by Eq. (2)

**  **update velocity by Eq. (3)

**  **randomly select rival and update velocity by Eq. (4)

****$\frac{3}{5}\cdot v_{n,i,d}^{t}+c_{1}r_{1,n,i,d}^{t}(x_{n,i,d}^{best}-x_{n,i,d}^{t})+c_{2}r_{2,n,i,d}^{t}(x_{tribe}^{best}-x_{n,i,d}^{t})$

**  if** $F_{n}^{best}<F_{rival}^{best}$ **then**

**    **update retreat factor by Eq. (2)

****$v_{n,i,d}^{t+1}=v_{n,i,d}^{t+1}-c_{3}r_{3,n,i,d}^{t}(x_{n,i,d}^{t}-x_{rival}^{best})$

**  **update human position $x_{n,i,d}^{t+1}$ by Eq. (6)

****$x_{n,i,d}^{t+1}=x_{n,i,d}^{t}+v_{n,i,d}^{t+1}$

**  **update human position $x_{n,i,d}^{t+1}$ by Eq. (8)

****$x_{new}=x_{rival}^{best}+x_{rival}^{best}\cdot t(iter)$

** if** human position is out of bound **then**

****$v_{n,i,d}^{t+1}=-v_{n,i,d}^{t+1}$

** **compute the fitness *F*

**  if** $F<F_{rival}^{best}$ **then**

**  **$x_{rival}^{best}$ =*x*

****$F_{rival}^{best}=F$

**  if** $F<F_{best}$ **then**

**  **$x^{best}$ =*x*

****$F^{best}=F$

** **Retrieve the current position

** **iter = iter+1

**return** $x^{best}$,$F^{best}$

**Fig 1:**
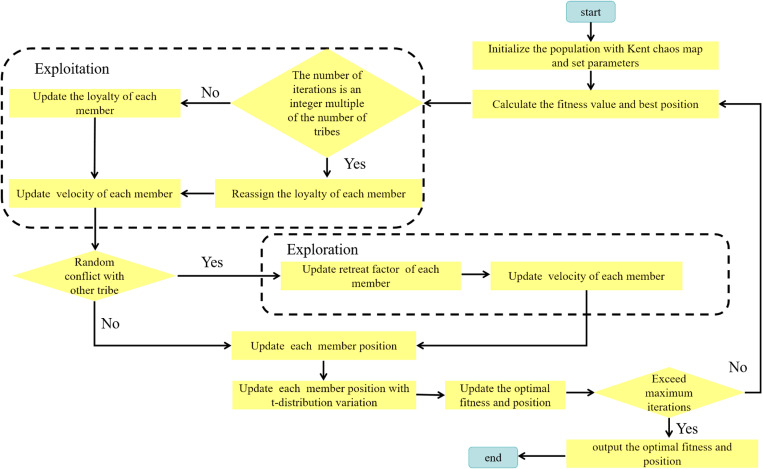
Flow chart of CTCMKT.

As can be seen, Fig 1 illustrates the flow chart of CTCMKT.

### Convergence analysis

This section elaborates on the theoretical convergence analysis of the CTCMKT algorithm. Meanwhile, effective theoretical analysis can further guide the design of CTCMKT application parameters. Assuming p^*^ is the individual’s optimal solution, f^*^ is the population’s optimal solution, and x^*^ is the global optimal point. In order to conduct analysis, it is assumed that p^*^ = f^*^ = x^*^ during the convergence process. Therefore, the CTCMKT speed update equation can be written as Eq.(9).


$$\begin{array}{cc} v^{k+1}=\; & \frac{3}{5}\cdot v^{k}+c_{1}r_{1,k}(p^{*}-x^{k})+c_{2}r_{2,k}(f^{*}-x^{k})+c_{3}r_{3,k}(x^{k}-x^{*})\\ =\; & \frac{3}{5}\cdot V^{k}+(c_{1}r_{1,k}+c_{2}r_{2,k}-c_{3}r_{3,k})*(x^{*}-x^{k}) \end{array}$$ (9)

Among them, $v^{k+1}$ represents the individual’s velocity at the k + 1 iteration, and


$$\mathrm{\backslash [}r_{j,k}=\sin(\frac{\pi }{2}r_{j,k-1}),j\in \{1,2,3\}\mathrm{\backslash ]}$$ (10)

The positional offset between the k-th iteration and the optimal solution is *s*^k^ as shown in Eq. (11).


$$\mathrm{\backslash [}s^{k}=x^{k}-x^{*}\mathrm{\backslash ]}$$ (11)

Then Eq.(9) can be rewritten as Eq.(12).


$$\mathrm{\backslash [}v^{k+1}=\frac{3}{5}\cdot v^{k}-(c_{1}r_{1,k}+c_{2}r_{2,k}-c_{3}r_{3,k})s^{k}=\frac{3}{5}\cdot v^{k}-c_{k}s^{k}\mathrm{\backslash ]}$$ (12)

And *c*_*k*_* = c*_*1*_ *r*_*1,k *_*+ c*_*2*_ *r*_*2,k *_*− c*_*3*_ *r*_*3,k*_. Substituting $s^{k+1}-s^{k}=x^{k+1}-x^{k}=v^{k+1}$ into Eq.(12) to eliminate the velocity term, then


$$\mathrm{\backslash [}s^{k+1}=\frac{3}{5}\cdot (s^{k}-s^{k-1})+(1-c_{k})s^{k}=(1.6-c^{k})s^{k}-0.6\cdot s^{k-1}\mathrm{\backslash ]}$$ (13)

The characteristic root equation of the difference equation obtained from this is shown in Eq.(14).


$$\mathrm{\backslash [}\omega^{2}-(1.6-c_{k})\omega +0.6=0\mathrm{\backslash ]}$$ (14)

If *c*_k_ is a constant, then the solution of the characteristic root equation should be as shown in Eq. (15).


$$\mathrm{\backslash [}\omega_{1,2}=\frac{(1.6-c_{k})\pm \sqrt{{\left(1.6-c_{k}\right)}^{2}-2.4}}{2}\mathrm{\backslash ]}$$ (15)

To ensure that CTCMKT can be in a stable state, the modulus of the eigenvalues must be less than 1, i.e., (1.6-*c*_k_)^2^ < 2.4 and ǀ1.6- *c*_k_ǀ < 2.4, so *c*_k_∈ (0, 3.15). Because of *r*_*j, k*_∈[0,1], let us only consider extreme situations. If *r*_1, k_ = 1, *r*_2, k_ = 1 and *r*_3, k_ = 0, so 0<(*c*_1_ + *c*_2_)<3.15, where *c*_3_ is an unstable perturbation term; if *c*_3_ is greater than 0, the system will exhibit instability at some point. As mentioned earlier, if *c*_3_> (*c*_1_*r*_1_ +* c*_2_*r*_2_−3.15)/*r*_3_, then the system will experience an increase in disturbance. So when adjusting parameters, *c*_1_ and *c*_2_ should first satisfy the condition of less than 3.15, and then *c*_3_ gradually increase from a very small positive number, in order to obtain the most ideal global optimization ability in different applications.

### Sensitivity analysis

Since the CTCMKT algorithm incorporates two strategies based on the original algorithm, it is necessary to analyze the selection of these strategies before comparing it with other algorithms. Among them, CTCMK represents the algorithm integrated with Kent chaotic mapping, and CTCMT represents the algorithm with *t*-distribution mutation. 23 basic test functions are selected as the test objects.

**Fig 2:**
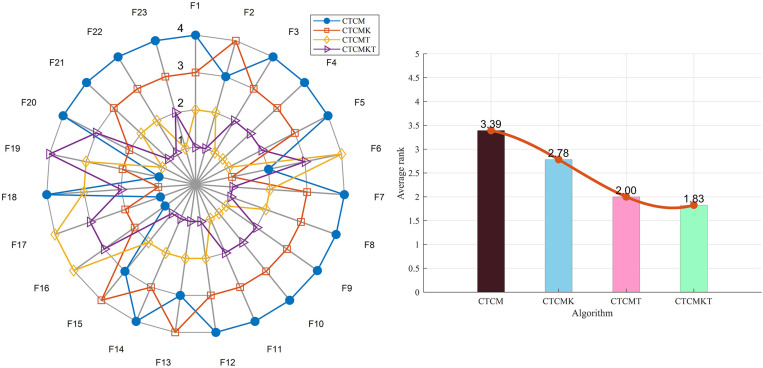
(a) Radar chart of 4 algorithms, (b) The sorting diagram of 4 algorithms.

The average optimal values of 20 tests are selected for comparison in the test results, and the results are shown in Table 1. At the same time, the radar chart and ranking chart are shown in Fig 2. In detail, compared with CTCM, the CTCMK algorithm shows a slight improvement in the performance of most functions, and its performance is only slightly worse in functions *F*_2_, *F*_13_, *F*_15_ - *F*_17_, and *F*_19_. The CTCMT algorithm shows a significant performance improvement compared to CTCM. However, its performance is slightly worse on functions *F*_6_, *F*_16_, *F*_17_, and *F*_19_. The CTCMKT algorithm with the combined strategy shows a slight improvement in the optimization – seeking ability compared to the CTCMT algorithm, and a significant improvement compared to the CTCM and CTCMK algorithms. As can be seen from the ranking chart, the rankings of the four algorithms are 3.39, 2.78, 2.00, and 1.83 respectively. In summary, the CTCMKT algorithm with the combined strategy has significantly improved in terms of optimization – seeking ability. In addition, it has also shown obvious enhancements in convergence speed and stability on most functions.

**Table 1:** Sensitivity analysis results for *F*_1_ to *F*_23_.

*F*	CTCM	CTCMK	CTCMT	CTCMKT
*F*_1_	1.14419E-19	2.2024E-20	2.7408E-246	3.3423E-249
*F*_2_	2.56338E-09	1.26902E-07	1.3073E-122	4.2035E-126
*F*_3_	300.3650303	260.1747553	1.724E-236	1.8028E-234
*F*_4_	8.590797467	8.205839761	1.375E-121	2.4189E-120
*F*_5_	42.49387204	39.59128224	26.83707222	26.87467612
*F*_6_	5.4898E-19	1.04283E-19	0.644141069	0.531228965
*F*_7_	0.105749424	0.098455391	9.07908E-05	8.88539E-05
*F*_8_	−6210.54784	−6753.381121	−9302.330834	−9546.563855
*F*_9_	43.12669134	31.10675802	0	0
*F*_10_	4.196129788	4.127576184	4.44089E-16	4.44089E-16
*F*_11_	0.027033147	0.016918265	0	0
*F*_12_	3.13294478	2.330086519	0.045417094	0.040096406
*F*_13_	9.973141623	12.7758071	1.767757092	1.717371569
*F*_14_	3.309902317	1.937028039	0.998004162	0.998003842
*F*_15_	0.000331244	0.000371814	0.000322459	0.000316135
*F*_16_	−1.031628453	−1.031628453	−1.03162655	−1.031626577
*F*_17_	0.397887358	0.397887358	0.397964512	0.397928242
*F*_18_	8.4	3	3.000011392	3.00001116
*F*_19_	−3.862782148	−3.862782148	−3.862590876	−3.86252074
*F*_20_	−3.286327235	−3.310105859	−3.313832882	−3.291992312
*F*_21_	−4.149751131	−8.89602484	−10.1531965	−10.15319658
*F*_22_	−5.218562576	−8.943958879	−10.40282051	−10.4028212
*F*_23_	−5.73183176	−7.826599198	−10.53628704	−10.53628511

## Experimental analysis

### Simulation environment and parameter settings

The simulation platform runs on a Windows 11 – based computer equipped with a 12th Gen Intel(R) Core (TM) i7 - 1260P 2.10GHz CPU, 16GB memory, and integrated graphics card. All algorithms are implemented in MATLAB R2023a. For the test function, the population size is configured as 40, the dimension is set at 20, the number of iterations is set to 1000, and all algorithm results represent the average of 30 runs. According to Chen et al.‘s research, *c*_1_, *c*_2_, and *c*_3_ are set to 2.0, 1.0, and 0.1, respectively. For all test sets, eight algorithms will be compared, including WOA [12], PSO [37], grey wolf optimizer (GWO) [13], differential evolution algorithm (DE) [38], beluga whale optimization algorithm (BWO) [39], goose algorithm (GOOSE) [40], CTCM [34], harris hawks optimization (HHO) [41], damping multi-verse optimizer (DMVO) [42] and CTCMKT.

### CEC2021 test functions

In this section, the CEC2021 function set will be used as a test set. The CEC2021 function set includes 10 single-objective functions, which are classified into the following categories: ‌Single-peak function (*f*_1_): This type of function usually has only one global minimum point. ‌Basic functions (*f*_2_-*f*_4_): These functions have different characteristics, and which are utilized for evaluating the performance of the algorithm under different conditions. ‌Hybrid functions (*f*_5_-*f*_7_): Combines multiple function characteristics to assess the performance in complex environments. ‌Combination function (*f*_8_-*f*_10_): These functions possess numerous local minimum points, serving to evaluate the algorithm’s performance within multi – peak environments. The CEC2021 test function defines the same search range, that is, the interval [−100, 100]. Min, std, avg, median, worse represent the optimal value, standard deviation, average value, median value and worst value, respectively.

The relevant statistical results of all algorithms solving formulas *f*_1_ to *f*_4_ are presented in Table 2. For *f*_1_, the optimal value, standard deviation, and average value of the CTCMKT have an absolute crushing advantage over the basic CTCM. Compared to other algorithms, the CTCMKT also ranks first, and which has both a smaller standard deviation and the best optimal value. It demonstrates that the CTCMKT possesses powerful global and local search abilities, enabling it to acquire the optimal solution efficiently. For *f*_2_, *f*_3_ and *f*_4_, CTCMKT can precisely identify the exact global optimization solution, and the performance of the optimal value, standard deviation, average value, and worst value are better than that of the basic CTCM and the remaining algorithms. It clarifies that CTCMKT has robust global optimization ability, allowing it to reach the optimal solution while avoiding being ensnared by local optimal solutions. The test results of *f*_1_ to *f*_4_ show that CTCMKT has perfect strong global optimization ability and stability for unimodal functions and basic functions.

**Table 2:** Experimental statistical results for *f*_1_ to *f*_4_.

*f*	Results	CTCMKT	CTCM	WOA	PSO	GWO	DE	BWO	GOOSE	HHO	DMVO
*f*_1_	min	7.553E-292	8.508E-122	3.207E-176	1.026E-83	3.443E-133	4.353E-32	1.157E + 01	1.942E + 00	1.599E-208	6.944E + 02
	std	0.000E + 00	2.236E + 03	0.000E + 00	7.109E-03	2.415E-125	4.107E-31	6.432E + 01	6.503E + 02	0.000E + 00	3.119E + 03
	avg	1.045E-249	5.000E + 02	2.594E-164	1.621E-03	7.783E-126	4.120E-31	8.770E + 01	3.575E + 02	7.517E-187	4.501E + 03
	median	7.393E-261	3.686E-115	1.486E-170	2.366E-79	2.424E-128	2.771E-31	7.897E + 01	1.141E + 02	6.054E-196	3.187E + 03
	worse	1.334E-248	1.000E + 04	5.011E-163	3.182E-02	9.395E-125	1.728E-30	2.675E + 02	2.724E + 03	1.480E-185	9.564E + 03
*f*_2_	min	0.000E + 00	1.874E-01	0.000E + 00	3.477E + 00	0.000E + 00	0.000E + 00	1.294E-04	1.852E-04	0.000E + 00	1.021E + 01
	std	0.000E + 00	1.798E + 02	4.397E + 02	1.968E + 02	2.683E + 00	4.911E-02	1.139E-03	6.770E + 02	0.000E + 00	2.499E + 02
	avg	0.000E + 00	1.837E + 02	1.831E + 02	3.091E + 02	1.076E + 00	4.684E-02	1.327E-03	1.162E + 03	0.000E + 00	4.532E + 02
	median	0.000E + 00	2.191E + 02	0.000E + 00	3.411E + 02	9.095E-13	6.245E-02	1.059E-03	1.219E + 03	0.000E + 00	4.150E + 02
	worse	0.000E + 00	5.175E + 02	1.413E + 03	7.372E + 02	1.072E + 01	1.249E-01	4.239E-03	2.001E + 03	0.000E + 00	8.717E + 02
*f*_3_	min	0.000E + 00	3.980E + 00	0.000E + 00	7.960E + 00	0.000E + 00	1.087E + 01	2.952E-05	1.990E + 00	0.000E + 00	7.967E + 00
	std	0.000E + 00	7.381E + 00	0.000E + 00	7.032E + 00	1.296E + 01	2.078E-15	5.289E-04	1.947E + 02	0.000E + 00	6.613E + 00
	avg	0.000E + 00	1.418E + 01	0.000E + 00	1.969E + 01	2.554E + 01	1.087E + 01	5.836E-04	2.168E + 02	0.000E + 00	2.438E + 01
	median	0.000E + 00	1.244E + 01	0.000E + 00	1.943E + 01	2.895E + 01	1.087E + 01	3.953E-04	2.145E + 02	0.000E + 00	2.369E + 01
	worse	0.000E + 00	2.985E + 01	0.000E + 00	3.275E + 01	4.269E + 01	1.087E + 01	2.026E-03	4.788E + 02	0.000E + 00	3.961E + 01
*f*_4_	min	0.000E + 00	4.945E-02	0.000E + 00	4.648E-01	0.000E + 00	5.752E-01	1.218E-13	5.436E-01	0.000E + 00	4.997E-01
	std	0.000E + 00	9.034E-01	5.619E-02	4.792E-01	4.519E-01	9.669E-02	5.365E-11	9.113E + 00	0.000E + 00	4.738E-01
	avg	0.000E + 00	1.064E + 00	1.818E-02	9.716E-01	3.780E-01	7.484E-01	3.396E-11	1.151E + 01	0.000E + 00	1.131E + 00
	median	0.000E + 00	8.473E-01	0.000E + 00	8.539E-01	1.790E-01	7.584E-01	1.177E-11	1.115E + 01	0.000E + 00	1.160E + 00
	worse	0.000E + 00	3.387E + 00	2.360E-01	2.631E + 00	1.358E + 00	9.487E-01	2.193E-10	2.770E + 01	0.000E + 00	2.368E + 00

The test results of all algorithms for functions *f*_5_ - *f*_10_ are presented in Table 3. For *f*_5_, *f*_7_, *f*_9_, and *f*_10,_ the CTCMKT algorithms have the best statistical data, include optimal value, average value, and standard deviation. This indicates that compared with other algorithms, the CTCMKT algorithm has better optimization solution solving ability and stability, and which can better escape from the local optimization solution. For *f*_6_ and *f*_8_, CTCMKT achieved accurate optimal solutions, and which showed excellent performance in terms of global solving ability and stability. And the modified CTCMKT exceeds the basic CTCM algorithm in terms of stability and global optimization. By comparing the solution results of all algorithms for *f*_5_-*f*_10_, it has been discovered that the CTCMKT algorithm showcases extraordinary stability and the proficiency to identify the global optimization solution during the resolution of complex problems.

**Table 3:** Experimental statistical results for *f*_5_ to *f*_10._

*f*	Results	CTCMKT	CTCM	WOA	PSO	GWO	DE	BWO	GOOSE	HHO	DMVO
*f*_5_	min	1.059E-278	4.180E-14	1.714E-168	2.212E + 01	2.181E-55	9.310E-32	2.396E-01	6.387E + 01	3.488E-210	1.331E + 02
	std	0.000E + 00	2.170E + 02	1.763E-19	2.650E + 02	8.966E-01	8.542E-02	6.527E + 00	2.109E + 03	0.000E + 00	2.618E + 02
	avg	6.812E-220	2.381E + 02	3.942E-20	4.720E + 02	2.359E-01	4.163E-02	5.313E + 00	2.823E + 03	1.099E-180	5.316E + 02
	median	2.485E-246	1.959E + 02	8.481E-143	4.355E + 02	3.657E-39	8.323E-30	2.838E + 00	2.708E + 03	1.423E-194	4.853E + 02
	worse	1.362E-218	9.170E + 02	7.884E-19	1.212E + 03	3.980E + 00	2.081E-01	2.208E + 01	7.426E + 03	2.198E-179	1.054E + 03
*f*_6_	min	0.000E + 00	7.483E-01	2.874E-04	3.981E-01	1.998E-02	6.005E-02	2.808E-03	2.844E-02	0.000E + 00	1.678E + 00
	std	0.000E + 00	5.732E + 00	1.636E + 00	2.692E + 01	1.078E + 00	7.456E-02	8.386E-03	2.310E + 02	8.548E-05	5.722E + 01
	avg	0.000E + 00	3.631E + 00	5.310E-01	8.435E + 00	5.795E-01	1.399E-01	1.657E-02	2.294E + 02	2.509E-05	5.013E + 01
	median	0.000E + 00	1.495E + 00	3.008E-02	1.385E + 00	1.222E-01	1.161E-01	1.524E-02	2.459E + 02	3.198E-10	1.466E + 01
	worse	0.000E + 00	2.281E + 01	7.305E + 00	1.209E + 02	4.796E + 00	2.996E-01	3.289E-02	5.837E + 02	3.783E-04	1.357E + 02
*f*_7_	min	4.766E-287	7.752E-01	2.882E-04	9.764E-01	7.538E-04	2.152E-03	3.147E-01	2.971E + 00	1.354E-230	2.730E + 01
	std	0.000E + 00	9.787E + 01	2.068E-01	1.564E + 02	1.488E-01	6.931E-02	4.487E + 00	2.862E + 03	4.168E-06	8.881E + 01
	avg	6.572E-258	4.784E + 01	8.965E-02	1.292E + 02	4.420E-02	2.823E-02	4.010E + 00	3.692E + 03	1.308E-06	1.139E + 02
	median	4.016E-271	1.765E + 01	1.594E-02	4.048E + 01	1.076E-02	1.052E-02	2.471E + 00	3.230E + 03	1.884E-10	7.330E + 01
	worse	1.314E-256	4.433E + 02	8.302E-01	4.628E + 02	6.752E-01	3.212E-01	1.453E + 01	8.857E + 03	1.843E-05	2.987E + 02
*f*_8_	min	0.000E + 00	2.203E + 01	0.000E + 00	2.033E + 01	0.000E + 00	0.000E + 00	1.887E-04	1.341E-04	0.000E + 00	2.145E + 01
	std	0.000E + 00	7.401E + 01	0.000E + 00	1.124E + 02	0.000E + 00	0.000E + 00	2.304E-03	7.333E + 02	0.000E + 00	3.026E + 02
	avg	0.000E + 00	8.673E + 01	0.000E + 00	1.169E + 02	0.000E + 00	0.000E + 00	2.672E-03	1.067E + 03	0.000E + 00	2.316E + 02
	median	0.000E + 00	4.228E + 01	0.000E + 00	5.796E + 01	0.000E + 00	0.000E + 00	1.771E-03	1.437E + 03	0.000E + 00	5.325E + 01
	worse	0.000E + 00	2.633E + 02	0.000E + 00	3.550E + 02	0.000E + 00	0.000E + 00	9.455E-03	1.891E + 03	0.000E + 00	8.501E + 02
*f*_9_	min	6.947E-278	8.882E-15	7.650E-184	8.882E-15	1.334E-96	8.882E-15	9.456E-03	5.094E-03	2.031E-223	8.766E-02
	std	0.000E + 00	2.462E + 00	5.372E-15	2.255E + 00	1.986E-15	0.000E + 00	1.512E-02	3.958E + 01	0.000E + 00	1.440E + 00
	avg	1.800E-254	1.379E + 00	4.885E-15	1.090E + 00	8.438E-15	8.882E-15	2.906E-02	6.616E + 01	2.785E-196	7.349E-01
	median	2.232E-263	1.776E-14	4.441E-15	8.882E-15	8.882E-15	8.882E-15	2.714E-02	8.268E + 01	1.112E-203	2.675E-01
	worse	3.322E-253	6.009E + 00	1.776E-14	6.311E + 00	8.882E-15	8.882E-15	5.614E-02	1.012E + 02	5.556E-195	4.944E + 00
*f*_10_	min	1.327E-278	3.625E + 01	7.448E-03	4.811E + 01	4.839E + 01	4.799E + 01	2.539E-02	1.950E-02	6.212E-207	4.828E + 01
	std	0.000E + 00	1.518E + 01	2.748E-02	1.420E + 01	8.032E + 00	9.990E-02	1.856E-02	5.328E + 01	1.405E-04	4.230E-01
	avg	4.370E-252	5.872E + 01	6.996E-02	5.864E + 01	5.262E + 01	4.807E + 01	5.522E-02	7.061E + 01	7.277E-05	4.889E + 01
	median	1.863E-264	4.991E + 01	7.414E-02	4.916E + 01	5.019E + 01	4.802E + 01	5.409E-02	8.637E + 01	4.946E-07	4.875E + 01
	worse	8.739E-251	9.516E + 01	1.180E-01	8.274E + 01	7.608E + 01	4.839E + 01	8.906E-02	1.470E + 02	4.669E-04	4.996E + 01

**Fig 3:**
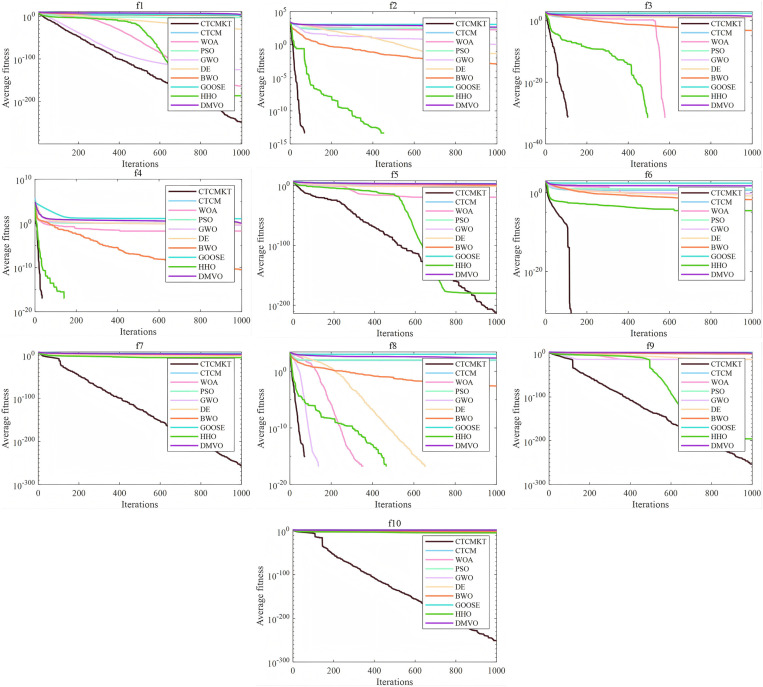
Convergence graphs of CEC2021 test functions.

Fig 3 presents the convergence curve graphs of the algorithms for CEC2021. The convergence curve effectively evaluates algorithm’s ability to converge and calculation accuracy in solving the optimal solution of the function. For *f*_1_ to *f*_4_, the CTCMKT algorithm effectively avoids the problems of falling into local optimal solutions and premature convergence by initializing the population through chaotic mapping and updating the position through *t*-distribution mutation, thereby improving the algorithm’s solution capability. And related parameters include standard deviation and average value, which are better than other algorithms as shown in Table 2. In Fig 3, it can be seen that the convergence speed and calculation precision of the enhanced CTCMKT are superior to those of the basic CTCM. This implies that the CTCMKT holds more powerful search and optimization capacities. For *f*_5_ to *f*_7_, *f*_9_ and *f*_10_, all performance evaluation indicators of the CTCMKT algorithm are better than those of other algorithms, and the convergence speed is also the fastest. For *f*_8_, WOA, GWO, DE and HHO can be comparable to CTCMKT in terms of statistical values, but the convergence speed of CTCMKT algorithm still ranks first. Compared with the remaining algorithms, the CTCMKT has an absolute advantage in both indicators and convergence speed. By integrating Kent chaotic mapping with *t*-distribution mutation, the diversity within the population and the global search capacity are efficiently boosted. As a result, CTCMKT achieve a quicker convergence rate and a higher level of calculation precision.

**Fig 4:**
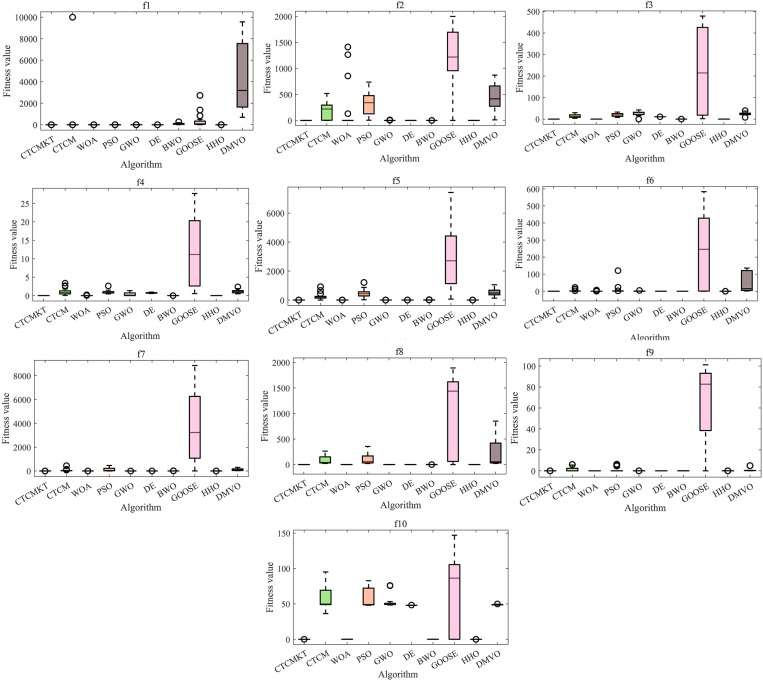
ANOVA tests for CEC2021.

The ANOVA test graphs for CEC2021 test set are shown in Fig 4. For solving equation optimization problems, the most important evaluation indicator is the standard deviation. A small standard deviation indicates that the algorithm not only has excellent global optimization capabilities but also high stability. Through Kent chaotic mapping and t – distribution mutation, the exploration and exploitation abilities of the CTCM algorithm are enhanced. As a result, the algorithm attains a more rapid convergence speed and higher computational precision. For *f*_1_ to *f*_10_, compared to the CTCM, the CTCMKT with the joint strategy has a smaller standard deviation, indicating that the joint strategy can effectively improve the stability of the algorithm and the ability of global optimization. In comparison to the other eight algorithms, the CTCMKT stands out with not only the minimum standard deviation, but also the most favorable optimal value, average value, and worst value. This clearly demonstrates the algorithm’s robust stability and significant advantages.

In Table 4, the results of the Wilcoxon rank sum test are presented. If the value is less than 0.05, it means that there is a significant difference between CTCMKT and the comparison algorithm in this function. It can be seen from the data in the table that basically all *p* values are less than 0.05. Therefore, it can be shown that, for CEC2021, there are significant differences between CTCMKT and other functions.

**Table 4:** The Wilcoxon results for CEC2021.

*f*	CTCM	WOA	PSO	GWO	DE	BWO	GOOSE	HHO	DMVO
*f*_1_	6.796E-08	6.796E-08	6.796E-08	6.796E-08	6.796E-08	6.796E-08	6.796E-08	6.796E-08	6.796E-08
*f*_2_	7.992E-09	2.077E-03	8.007E-09	1.604E-04	9.084E-06	8.007E-09	8.007E-09	1.000E + 00	8.007E-09
*f*_3_	8.007E-09	1.000E + 00	8.007E-09	3.505E-07	5.621E-09	8.007E-09	8.007E-09	1.000E + 00	8.007E-09
*f*_4_	8.007E-09	8.063E-02	8.007E-09	1.105E-06	8.007E-09	8.007E-09	8.007E-09	1.000E + 00	8.007E-09
*f*_5_	6.796E-08	6.796E-08	6.796E-08	6.796E-08	6.700E-08	6.796E-08	6.796E-08	6.796E-08	6.796E-08
*f*_6_	8.007E-09	8.007E-09	8.007E-09	8.007E-09	8.007E-09	8.007E-09	8.007E-09	1.105E-06	8.007E-09
*f*_7_	6.796E-08	6.796E-08	6.796E-08	6.796E-08	6.796E-08	6.796E-08	6.796E-08	6.796E-08	6.796E-08
*f*_8_	8.007E-09	1.000E + 00	8.007E-09	1.000E + 00	1.000E + 00	8.007E-09	8.007E-09	1.000E + 00	8.007E-09
*f*_9_	5.735E-08	5.727E-08	2.955E-08	1.127E-08	8.007E-09	6.796E-08	6.796E-08	6.796E-08	6.796E-08
*f*_10_	6.796E-08	6.796E-08	6.796E-08	6.796E-08	6.796E-08	6.796E-08	6.796E-08	6.796E-08	6.796E-08

**Fig 5:**
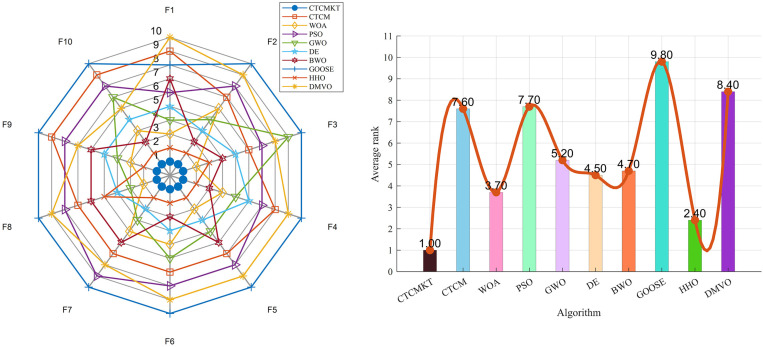
(a) Radar chart of these algorithms (CEC2021), (b) The sorting diagram of these algorithms (CEC2021).

The radar chart and sorting diagram of the CEC2021 test function are shown in Fig 5. (a)-(b). Radar chart is a graphical method for displaying multidimensional data in two dimensions. It maps data from multiple dimensions to axes starting from the center point, where each axis represents a variable. By connecting the data points on each axis to form a polygon, the performance of different categories in each dimension can be intuitively compared. It can be seen from the figure that CTCMKT (blue circle) ranks first in the test results of all algorithms, and this result can also be confirmed from the average ranking chart. This reveals that the CTCMKT improved by the joint strategy is more excellent than other algorithms in global optimization ability and stability.

### 23 benchmark functions

In this section, the 23 benchmark functions will be tested. And the 23 benchmark functions are shown in Table 5, where *F*_1_-*F*_7_ are unimodal test functions, *F*_8_-*F*_13_ are multimodal test functions, and *F*_14_-*F*_23_ are fixed-dimension multimodal functions.

**Tabel 5:** 23 benchmark functions.

Benchmark functions	Range	*f*_min_
$f_{1}(x)=\sum {\mathrm{\backslash nolimits}}_{i=1}^{D}x_{i}^{2}$	[-100,100]	0
$f_{2}(x)=\sum {\mathrm{\backslash nolimits}}_{i=1}^{D}\left|x_{i}\right|+\prod {\mathrm{\backslash nolimits}}_{i=1}^{D}\left|x_{i}\right|$	[-10,10]	0
$f_{3}(x)=\sum {\mathrm{\backslash nolimits}}_{i=1}^{D}{\left(\sum {\mathrm{\backslash nolimits}}_{j=1}^{i}x_{j}\right)}^{2}$	[-100,100]	0
$f_{4}(x)=\max_{i}\{\left|x_{i}\right|\}$	[-100,100]	0
$f_{5}(x)=\sum {\mathrm{\backslash nolimits}}_{i=1}^{D-1}\left[100{\left(x_{i+1}-x_{i}^{2}\right)}^{2}+{\left(x_{i}-1\right)}^{2}\right]$	[-30,30]	0
$f_{6}(x)=\sum {\mathrm{\backslash nolimits}}_{i=1}^{D}{\left(\left\lfloor x_{i}+0.5\right\rfloor \right)}^{2}$	[-100,100]	0
$f_{7}(x)=\sum {\mathrm{\backslash nolimits}}_{i=1}^{D}ix_{i}^{2}+random[0,1)$	[-1.28,1.28]	0
$f_{8}(x)=\sum {\mathrm{\backslash nolimits}}_{i=1}^{D}x_{i}^{2}\sin(\sqrt{\left|x_{i}\right|})$	[-500,500]	−418.9829D
$f_{9}(x)=\sum {\mathrm{\backslash nolimits}}_{i=1}^{D}[x_{i}^{2}-10\cos(2\pi x_{i})+10]$	[-5.12,5.12]	0
$\begin{array}{c} f_{10}(x)=-20exp(-0.2\sqrt{\frac{1}{D}\sum {\mathrm{\backslash nolimits}}_{i=1}^{D}x_{i}^{2}}-\exp(\frac{1}{D}\sum {\mathrm{\backslash nolimits}}_{i=1}^{D}\cos(2\pi x_{i})nonumber\\ +20+e \end{array}$	[-32,32]	0
$f_{11}(x)=\sum {\mathrm{\backslash nolimits}}_{i=1}^{D}\frac{{x_{i}}^{2}}{4000}-\prod {\mathrm{\backslash nolimits}}_{i=1}^{D}\cos(\frac{x_{i}}{\sqrt{i}})+1$	[-600,600]	0
$\begin{array}{c} f_{12}(x)=\frac{\pi }{D}\{10\sin^{2}(\pi y_{1})+\sum_{i=1}^{D-1}{\left(y_{i}-1\right)}^{2}[1+10\sin^{2}(\pi y_{i+1})]\\ +(y_{D}-1{)}^{2}\}+\sum_{i=1}^{D}\mu (x_{i},10,100,4)\\ y_{i}=1+\frac{x_{i}+1}{4}\\ \mu (x_{i},a,k,m)=\{\begin{array}{ccc} {}*20ck{\left(x_{i}-a\right)}^{m} & & x_{i}>a\\ 0 & & -a\leq x_{i}\leq a\\ k{\left(-x_{i}-a\right)}^{m} & & x_{i}<-a \end{array} \end{array}$	[-50,50]	0
$\begin{array}{c} f_{13}(x)=0.1\{\sin2(3\pi x1+g(x))+\sum_{i=1}^{D}\mu (x_{i},5,100,4)\\ g(x)=\sum_{i=1}^{D-1}{\left(x_{i}-1\right)}^{2}[1+\sin^{2}(3\pi x_{i}+1)]\\ +(x_{D}-1{)}^{2}[1+\sin^{2}(2\pi x_{D})] \end{array}$	[-50,50]	0
$f_{14}(x)={\left(\frac{1}{500}+\sum_{j=1}^{25}\frac{1}{j+\sum_{i=1}^{2}{\left(x_{i}-a_{ij}\right)}^{6}}\right)}^{-1}$	[-65,65]	1
$f_{15}(x)=\sum_{i=1}^{11}{\left[a_{i}-\frac{x_{1}(b_{i}^{2}+b_{1}x_{2})}{b_{i}^{2}+b_{1}x_{3}+x_{4}}\right]}^{2}$	[-5,5]	0.1484
$f_{16}(x)=4x_{1}^{2}-2.1x_{1}^{4}+\frac{1}{3}x_{1}^{6}+x_{1}x_{2}-4x_{2}^{2}+4x_{2}^{4}$	[-5,5]	−1.0316
$\begin{array}{c} f_{17}(x)={\left(x_{2}-\frac{5.1}{4\pi^{2}}x_{1}^{2}+\frac{5}{\pi }x_{1}-6\right)}^{2}+10\left(1-\frac{1}{8\pi }\right)\cos x_{1}+10 \end{array}$	[-5,5], [10,15]	0.3979
$\begin{array}{c} f_{18}(x)=[1+(x_{1}+x_{2}+1{)}^{2}(19-14x_{1}+3x_{1}^{2}-14x_{2}+6x_{1}x_{2}+3x_{2}^{2}]\\ \times [30+(2x_{1}-3x_{2}{)}^{2}(18-32x_{1}+12x_{1}^{2}+48x_{2}-36x_{1}x_{2}+27x_{2}^{2})] \end{array}$	[-2,2]	3
$f_{19}(x)=-\sum_{i=1}^{4}c_{i}\exp\left(-\sum_{j=1}^{3}a_{ij}{\left(x_{j}-pi_{j}\right)}^{2}\right)$	[0,1]	−3
$f_{20}(x)=-\sum_{i=1}^{4}c_{i}\exp\left(-\sum_{j=1}^{36}a_{ij}{\left(x_{j}-pi_{j}\right)}^{2}\right)$	[0,1]	−3
$f_{21}(x)=-\sum_{i=1}^{5}{\left[(X-a_{i}){\left(X-a_{i}\right)}^{T}+c_{i}\right]}^{-1}$	[0,10]	−1
$f_{22}(x)=-\sum_{i=1}^{7}{\left[(X-a_{i}){\left(X-a_{i}\right)}^{T}+c_{i}\right]}^{-1}$	[0,10]	−1
$f_{23}(x)=-\sum_{i=1}^{10}{\left[(X-a_{i}){\left(X-a_{i}\right)}^{T}+c_{i}\right]}^{-1}$	[0,10]	−1

The relevant statistical results of all algorithms for solving functions *F*_1_ to *F*_7_ are shown in Table 6. For *F*_1_ to *F*_4_, compared with other algorithms, CTCMKT algorithm ranks first in terms of optimal value, average value, standard deviation and worst value, which indicates that the CTCMKT algorithm has strong stability and can effectively avoid premature convergence. For *F*_5_ to *F*_7_, CTCMKT ranked fifth, fifth and ninth respectively, although the ranking is low, the average value is relatively small, which shows that the CTCMKT has stronger stability and optimization capabilities among several algorithms. Comprehensive consideration of functions *F*_1_ to *F*_7_ shows that the CTCMKT algorithm has strong search capability and accuracy in solving unimodal functions.

**Table 6:** Experimental statistical results for *F*_1_ to *F*_7._

*f*	Results	CTCMKT	CTCM	WOA	PSO	GWO	DE	BWO	GOOSE	HHO	DMVO
*F*_1_	min	5.185E-275	1.234E-23	3.035E-178	4.104E-02	3.158E-68	5.684E-09	7.916E-06	8.993E-04	3.867E-213	7.931E-02
	std	0.000E + 00	4.107E-20	5.810E-158	1.396E + 01	2.171E-65	3.775E-09	2.322E-04	4.878E-04	0.000E + 00	6.072E-02
	avg	2.706E-247	2.156E-20	1.299E-158	5.378E + 00	1.012E-65	1.081E-08	3.024E-04	1.652E-03	2.719E-187	2.232E-01
	median	2.928E-258	4.165E-21	2.577E-171	8.524E-01	2.559E-66	9.468E-09	2.785E-04	1.587E-03	2.856E-202	2.329E-01
	worse	5.411E-246	1.452E-19	2.598E-157	6.098E + 01	9.632E-65	2.056E-08	7.756E-04	2.473E-03	2.459E-186	3.061E-01
*F*_2_	min	1.708E-143	2.311E-12	2.732E-116	1.602E-01	2.811E-39	2.788E-06	3.279E-03	1.833E-01	2.361E-113	1.583E-01
	std	2.423E-128	2.047E-09	7.337E-106	2.499E + 00	2.268E-38	9.475E-07	3.907E-03	6.009E + 02	1.616E-98	7.885E-02
	avg	1.273E-128	1.107E-09	1.644E-106	2.493E + 00	2.215E-38	4.076E-06	9.661E-03	1.653E + 02	4.482E-99	3.272E-01
	median	1.446E-130	3.509E-10	7.714E-111	1.919E + 00	1.553E-38	3.893E-06	8.786E-03	4.406E-01	8.005E-104	3.133E-01
	worse	8.995E-128	8.780E-09	3.281E-105	1.202E + 01	9.677E-38	6.360E-06	1.717E-02	2.710E + 03	7.180E-98	4.753E-01
*F*_3_	min	1.501E-266	5.783E-01	3.753E + 03	1.159E + 02	2.283E-23	2.040E + 04	1.062E-02	1.708E-01	2.641E-181	1.451E + 01
	std	0.000E + 00	3.595E + 02	9.100E + 03	1.136E + 03	1.161E-17	4.428E + 03	9.562E-02	7.211E-01	1.200E-156	9.121E + 00
	avg	9.224E-233	1.495E + 02	1.647E + 04	9.746E + 02	6.040E-18	2.824E + 04	1.043E-01	8.309E-01	3.089E-157	2.652E + 01
	median	6.736E-246	9.192E + 00	1.654E + 04	5.785E + 02	3.772E-19	2.855E + 04	6.811E-02	5.035E-01	2.256E-169	2.625E + 01
	worse	1.842E-231	1.213E + 03	4.203E + 04	5.296E + 03	3.868E-17	3.648E + 04	3.716E-01	2.851E + 00	5.348E-156	4.376E + 01
*F*_4_	min	1.284E-136	5.445E + 00	1.892E-01	6.119E + 00	8.751E-18	4.003E + 00	1.077E-03	3.253E-02	9.378E-110	4.033E-01
	std	7.213E-119	1.523E + 00	3.045E + 01	2.405E + 00	4.056E-16	7.354E-01	1.310E-03	1.154E + 01	1.495E-96	2.922E-01
	avg	2.076E-119	8.499E + 00	3.957E + 01	1.130E + 01	3.601E-16	5.067E + 00	3.072E-03	1.157E + 01	4.991E-97	8.445E-01
	median	6.740E-127	8.461E + 00	4.673E + 01	1.160E + 01	2.405E-16	4.992E + 00	2.591E-03	1.351E + 01	3.467E-100	7.785E-01
	worse	3.108E-118	1.189E + 01	8.561E + 01	1.619E + 01	1.750E-15	7.215E + 00	6.113E-03	3.201E + 01	5.338E-96	1.484E + 00
*F*_5_	min	2.624E + 01	8.079E + 00	2.626E + 01	7.911E + 01	2.529E + 01	2.657E + 01	2.282E-05	2.604E + 01	4.281E-06	2.566E + 01
	std	2.759E-01	4.297E + 01	2.451E-01	4.059E + 02	7.278E-01	2.240E + 01	6.488E-04	6.013E + 01	5.579E-03	3.394E + 02
	avg	2.680E + 01	6.037E + 01	2.674E + 01	3.012E + 02	2.673E + 01	5.215E + 01	9.614E-04	7.119E + 01	3.609E-03	1.869E + 02
	median	2.685E + 01	6.242E + 01	2.675E + 01	1.977E + 02	2.655E + 01	4.855E + 01	8.588E-04	2.951E + 01	8.733E-04	7.392E + 01
	worse	2.717E + 01	1.345E + 02	2.710E + 01	1.950E + 03	2.798E + 01	1.008E + 02	2.475E-03	2.273E + 02	2.029E-02	1.520E + 03
*F*_6_	min	3.788E-01	5.553E-23	3.669E-03	1.691E-02	1.459E-05	3.942E-09	3.410E-02	7.285E-04	6.053E-08	1.540E-01
	std	1.805E-01	2.671E-20	5.058E-02	2.399E + 00	2.754E-01	6.464E-09	2.637E-01	4.919E-04	3.494E-05	5.147E-02
	avg	6.137E-01	1.628E-20	2.149E-02	1.620E + 00	3.623E-01	1.291E-08	3.605E-01	1.500E-03	3.048E-05	2.238E-01
	median	5.989E-01	3.651E-21	8.331E-03	8.517E-01	2.515E-01	1.087E-08	2.825E-01	1.542E-03	2.349E-05	2.042E-01
	worse	1.102E + 00	1.007E-19	2.339E-01	9.868E + 00	1.002E + 00	3.294E-08	8.660E-01	2.467E-03	1.313E-04	3.662E-01
*F*_7_	min	1.600E-05	4.888E-02	4.946E-05	4.120E-02	1.822E-04	2.364E-02	5.394E-05	1.956E-02	7.192E-06	4.812E-03
	std	6.121E-05	5.648E-02	8.225E-04	7.522E-02	3.493E-04	7.290E-03	1.219E-04	2.625E-02	5.782E-05	6.698E-03
	avg	8.381E-05	1.189E-01	1.082E-03	1.323E-01	6.416E-04	3.202E-02	2.011E-04	5.089E-02	5.706E-05	1.521E-02
	median	8.574E-05	1.053E-01	8.584E-04	1.120E-01	5.715E-04	2.931E-02	1.819E-04	4.597E-02	4.324E-05	1.454E-02
	worse	2.549E-04	2.606E-01	2.982E-03	3.297E-01	1.478E-03	5.249E-02	5.211E-04	1.193E-01	2.628E-04	2.946E-02

The test results of the functions *F*_8_ to *F*_13_ are shown in Table 7. For *F*_8_, the optimal value and standard deviation of CTCMKT algorithm can only be ranked at a medium level, and its stability is slightly worse than that of the other algorithms. For functions *F*_9_ to *F*_11_, the CTCMKT algorithm ranks first with a standard deviation of 0, and can find the exact optimization solution, which shows that the CTCMKT algorithm can find the global optimal solution, avoid falling into the local optimization solution, and possess strong stability. For functions *F*_12_ and *F*_13_, the CTCMKT algorithm ranks fifth and eighth, and data such as standard deviation are better than those of the basic CTCM algorithm. It also has relatively small standard deviation and optimal value, indicating that CTCMKT possess better search ability and stability. In general, the CTCMKT algorithm with a joint strategy has better global optimization capability and stability than CTCM, can avoid premature convergence, and possess a good effect in solving multimodal test functions.

**Table 7:** Experimental statistical results for *F*_8_ to *F*_13._

*f*	Results	CTCMKT	CTCM	WOA	PSO	GWO	DE	BWO	GOOSE	HHO	DMVO
*F*_8_	min	−1.135E + 04	−7.969E + 03	−1.257E + 04	−7.890E + 03	−7.185E + 03	−1.257E + 04	−1.257E + 04	−8.758E + 03	−1.257E + 04	−9.024E + 03
	std	1.068E + 03	7.256E + 02	1.576E + 03	9.209E + 02	8.001E + 02	3.330E + 02	2.980E + 00	7.967E + 02	9.750E + 01	6.173E + 02
	avg	−9.475E + 03	−6.495E + 03	−1.081E + 04	−6.520E + 03	−6.157E + 03	−1.240E + 04	−1.257E + 04	−7.133E + 03	−1.255E + 04	−7.714E + 03
	median	−9.474E + 03	−6.469E + 03	−1.106E + 04	−6.694E + 03	−6.308E + 03	−1.245E + 04	−1.257E + 04	−7.209E + 03	−1.257E + 04	−7.697E + 03
	worse	−7.893E + 03	−5.378E + 03	−7.163E + 03	−4.594E + 03	−3.877E + 03	−1.102E + 04	−1.256E + 04	−5.401E + 03	−1.213E + 04	−6.559E + 03
*F*_9_	min	0.000E + 00	2.192E + 01	0.000E + 00	2.924E + 01	0.000E + 00	5.381E + 01	7.784E-06	8.175E + 01	0.000E + 00	7.276E + 01
	std	0.000E + 00	8.987E + 00	0.000E + 00	1.271E + 01	1.883E + 00	7.095E + 00	1.134E-04	3.925E + 01	0.000E + 00	2.036E + 01
	avg	0.000E + 00	3.728E + 01	0.000E + 00	5.610E + 01	5.609E-01	6.554E + 01	1.444E-04	1.505E + 02	0.000E + 00	1.144E + 02
	median	0.000E + 00	3.933E + 01	0.000E + 00	5.675E + 01	0.000E + 00	6.767E + 01	9.078E-05	1.496E + 02	0.000E + 00	1.095E + 02
	worse	0.000E + 00	5.077E + 01	0.000E + 00	8.061E + 01	7.928E + 00	7.668E + 01	4.254E-04	2.490E + 02	0.000E + 00	1.554E + 02
*F*_10_	min	0.000E + 00	1.898E + 00	4.441E-16	2.311E + 00	7.550E-15	2.446E-05	3.197E-04	2.402E-02	0.000E + 00	1.297E-01
	std	0.000E + 00	7.512E-01	2.647E-15	1.064E + 00	3.281E-15	7.861E-06	1.832E-03	9.426E + 00	0.000E + 00	4.180E + 00
	avg	0.000E + 00	3.634E + 00	3.464E-15	4.399E + 00	1.359E-14	3.468E-05	3.240E-03	7.793E + 00	0.000E + 00	2.085E + 00
	median	0.000E + 00	3.786E + 00	3.997E-15	4.361E + 00	1.465E-14	3.393E-05	2.875E-03	7.757E-01	0.000E + 00	1.201E + 00
	worse	0.000E + 00	4.775E + 00	7.550E-15	6.280E + 00	2.176E-14	5.062E-05	7.335E-03	1.960E + 01	0.000E + 00	1.944E + 01
*F*_11_	min	0.000E + 00	1.443E-15	0.000E + 00	3.523E-02	0.000E + 00	1.278E-08	1.161E-05	7.506E-03	0.000E + 00	3.422E-01
	std	0.000E + 00	2.956E-02	1.512E-02	4.326E-01	3.068E-03	1.379E-07	3.240E-04	1.072E + 02	0.000E + 00	8.820E-02
	avg	0.000E + 00	1.965E-02	3.381E-03	5.937E-01	9.909E-04	2.012E-07	4.695E-04	1.524E + 02	0.000E + 00	4.965E-01
	median	0.000E + 00	9.189E-03	0.000E + 00	5.515E-01	0.000E + 00	2.173E-07	3.748E-04	1.995E + 02	0.000E + 00	4.870E-01
	worse	0.000E + 00	1.131E-01	6.761E-02	1.556E + 00	1.094E-02	4.964E-07	1.075E-03	2.822E + 02	0.000E + 00	6.342E-01
*F*_12_	min	1.149E-02	5.750E-01	4.236E-04	2.954E + 00	1.639E-06	1.057E-09	4.394E-05	3.983E + 00	3.270E-08	1.307E-03
	std	1.436E-02	1.300E + 00	4.084E-03	4.090E + 00	1.201E-01	1.221E-09	5.481E-04	5.190E + 00	7.116E-07	9.472E-01
	avg	4.470E-02	3.227E + 00	3.527E-03	6.897E + 00	4.996E-02	2.429E-09	4.296E-04	1.070E + 01	6.320E-07	9.567E-01
	median	4.432E-02	3.447E + 00	1.652E-03	6.170E + 00	2.475E-02	2.050E-09	1.935E-04	9.708E + 00	4.266E-07	5.837E-01
	worse	7.464E-02	5.276E + 00	1.590E-02	1.743E + 01	5.569E-01	5.158E-09	1.927E-03	2.435E + 01	3.051E-06	3.071E + 00
*F*_13_	min	7.054E-01	1.210E-02	1.215E-02	3.991E + 00	1.011E-01	2.486E-09	1.241E-07	2.391E-04	4.337E-09	1.537E-02
	std	6.730E-01	7.874E + 00	9.935E-02	8.396E + 00	1.276E-01	7.170E-09	7.485E-06	5.150E-03	1.549E-05	2.071E-02
	avg	1.971E + 00	1.140E + 01	9.498E-02	1.819E + 01	3.654E-01	1.014E-08	7.482E-06	3.451E-03	9.827E-06	5.381E-02
	median	2.102E + 00	1.108E + 01	5.089E-02	1.862E + 01	3.559E-01	7.755E-09	5.220E-06	6.042E-04	4.258E-06	5.298E-02
	worse	2.885E + 00	2.976E + 01	3.441E-01	3.052E + 01	6.623E-01	2.602E-08	2.921E-05	1.283E-02	6.856E-05	9.579E-02

Table 8 and Table 9 display the results obtained from testing fixed – dimension multimodal functions. For functions *F*_14_ to *F*_20_, the test results of all algorithms are at the same level, with small standard deviations and almost consistent optimal solutions, which indicates that CTCMKT has strong stability and global optimization capabilities and can avoid falling into local optimization solutions. For functions *F*_21_ to *F*_23_, all algorithms can search almost the same optimization solution, indicating that these algorithms are at a comparable level in finding the optimal solution. In terms of standard deviation, the CTCMKT algorithm has an absolute advantage over other algorithms, and the fluctuation of the optimal solution is very small, almost close to 0, indicating that the joint strategy has greatly improved the stability of CTCM. Comparing the optimal values and standard deviations of functions *F*_14_ to *F*_23_, the CTCMKT algorithm has better results, indicating that the combined strategy of Kent chaotic mapping and t-distribution mutation can effectively improve the global optimization ability of the algorithm and greatly improve the stability of the original algorithm.

**Table 8:** Experimental statistical results for *F*_14_ to *F*_18._

*f*	Results	CTCMKT	CTCM	WOA	PSO	GWO	DE	BWO	GOOSE	HHO	DMVO
*F*_14_	min	9.980E-01	9.980E-01	9.980E-01	9.980E-01	9.980E-01	9.980E-01	9.980E-01	1.992E + 00	9.980E-01	9.980E-01
	std	4.437E-01	2.910E + 00	8.143E-01	3.432E + 00	4.212E + 00	0.000E + 00	2.383E + 00	6.444E + 00	2.223E-01	5.677E-12
	avg	1.097E + 00	4.444E + 00	1.395E + 00	3.699E + 00	4.622E + 00	9.980E-01	4.580E + 00	1.025E + 01	1.048E + 00	9.980E-01
	median	9.980E-01	5.929E + 00	9.980E-01	1.495E + 00	2.982E + 00	9.980E-01	5.942E + 00	8.357E + 00	9.980E-01	9.980E-01
	worse	2.982E + 00	1.076E + 01	2.982E + 00	1.267E + 01	1.076E + 01	9.980E-01	7.023E + 00	2.290E + 01	1.992E + 00	9.980E-01
*F*_15_	min	3.086E-04	3.075E-04	3.082E-04	3.075E-04	3.075E-04	4.721E-04	3.224E-04	5.324E-04	3.075E-04	3.089E-04
	std	1.273E-05	1.621E-18	2.834E-04	4.455E-03	8.210E-03	1.277E-04	5.751E-05	8.658E-03	2.047E-04	9.240E-03
	avg	3.183E-04	3.075E-04	5.489E-04	1.526E-03	4.364E-03	6.770E-04	3.966E-04	5.756E-03	3.661E-04	6.610E-03
	median	3.130E-04	3.075E-04	4.933E-04	3.075E-04	3.075E-04	6.892E-04	3.884E-04	7.651E-04	3.191E-04	7.617E-04
	worse	3.492E-04	3.075E-04	1.621E-03	2.036E-02	2.036E-02	1.099E-03	5.549E-04	2.036E-02	1.234E-03	2.036E-02
*F*_16_	min	−1.032E + 00	−1.032E + 00	−1.032E + 00	−1.032E + 00	−1.032E + 00	−1.032E + 00	−1.032E + 00	−1.032E + 00	−1.032E + 00	−1.032E + 00
	std	1.830E-06	2.278E-16	2.791E-11	2.220E-16	5.487E-09	2.278E-16	6.068E-05	3.349E-01	5.865E-12	6.742E-08
	avg	−1.032E + 00	−1.032E + 00	−1.032E + 00	−1.032E + 00	−1.032E + 00	−1.032E + 00	−1.032E + 00	−8.684E-01	−1.032E + 00	−1.032E + 00
	median	−1.032E + 00	−1.032E + 00	−1.032E + 00	−1.032E + 00	−1.032E + 00	−1.032E + 00	−1.032E + 00	−1.032E + 00	−1.032E + 00	−1.032E + 00
	worse	−1.032E + 00	−1.032E + 00	−1.032E + 00	−1.032E + 00	−1.032E + 00	−1.032E + 00	−1.031E + 00	−2.155E-01	−1.032E + 00	−1.032E + 00
*F*_17_	min	3.979E-01	3.979E-01	3.979E-01	3.979E-01	3.979E-01	3.979E-01	3.979E-01	3.979E-01	3.979E-01	3.979E-01
	std	1.873E-05	0.000E + 00	3.312E-07	0.000E + 00	6.610E-07	0.000E + 00	2.951E-03	4.856E-11	8.862E-08	6.058E-08
	avg	3.979E-01	3.979E-01	3.979E-01	3.979E-01	3.979E-01	3.979E-01	4.003E-01	3.979E-01	3.979E-01	3.979E-01
	median	3.979E-01	3.979E-01	3.979E-01	3.979E-01	3.979E-01	3.979E-01	3.989E-01	3.979E-01	3.979E-01	3.979E-01
	worse	3.980E-01	3.979E-01	3.979E-01	3.979E-01	3.979E-01	3.979E-01	4.084E-01	3.979E-01	3.979E-01	3.979E-01
*F*_18_	min	3.000E + 00	3.000E + 00	3.000E + 00	3.000E + 00	3.000E + 00	3.000E + 00	3.303E + 00	3.000E + 00	3.000E + 00	3.000E + 00
	std	1.414E-05	1.321E + 01	1.109E-05	5.486E-16	6.765E-06	8.998E-16	8.601E + 00	6.037E + 00	1.432E-10	1.100E-06
	avg	3.000E + 00	1.245E + 01	3.000E + 00	3.000E + 00	3.000E + 00	3.000E + 00	1.461E + 01	4.350E + 00	3.000E + 00	3.000E + 00
	median	3.000E + 00	3.000E + 00	3.000E + 00	3.000E + 00	3.000E + 00	3.000E + 00	1.433E + 01	3.000E + 00	3.000E + 00	3.000E + 00
	worse	3.000E + 00	3.000E + 01	3.000E + 00	3.000E + 00	3.000E + 00	3.000E + 00	3.119E + 01	3.000E + 01	3.000E + 00	3.000E + 00

**Table 9:** Experimental statistical results for *F*_19_ to *F*_23._

*f*	Results	CTCMKT	CTCM	WOA	PSO	GWO	DE	BWO	GOOSE	HHO	DMVO
*F*_19_	min	−3.863E + 00	−3.863E + 00	−3.863E + 00	−3.863E + 00	−3.863E + 00	−3.863E + 00	−3.853E + 00	−3.863E + 00	−3.863E + 00	−3.863E + 00
	std	2.077E-04	2.278E-15	3.490E-03	2.278E-15	1.686E-03	2.278E-15	5.219E-02	1.050E-07	9.351E-04	1.177E-07
	avg	−3.863E + 00	−3.863E + 00	−3.861E + 00	−3.863E + 00	−3.862E + 00	−3.863E + 00	−3.764E + 00	−3.863E + 00	−3.862E + 00	−3.863E + 00
	median	−3.863E + 00	−3.863E + 00	−3.862E + 00	−3.863E + 00	−3.863E + 00	−3.863E + 00	−3.768E + 00	−3.863E + 00	−3.863E + 00	−3.863E + 00
	worse	−3.862E + 00	−3.863E + 00	−3.852E + 00	−3.863E + 00	−3.856E + 00	−3.863E + 00	−3.650E + 00	−3.863E + 00	−3.859E + 00	−3.863E + 00
*F*_20_	min	−3.321E + 00	−3.322E + 00	−3.322E + 00	−3.322E + 00	−3.322E + 00	−3.322E + 00	−3.267E + 00	−3.322E + 00	−3.320E + 00	−3.322E + 00
	std	5.018E-02	6.069E-02	8.863E-02	5.976E-02	9.177E-02	2.159E-15	1.496E-01	6.118E-02	6.485E-02	5.985E-02
	avg	−3.300E + 00	−3.268E + 00	−3.247E + 00	−3.274E + 00	−3.241E + 00	−3.322E + 00	−2.984E + 00	−3.262E + 00	−3.208E + 00	−3.274E + 00
	median	−3.320E + 00	−3.322E + 00	−3.321E + 00	−3.322E + 00	−3.263E + 00	−3.322E + 00	−3.017E + 00	−3.263E + 00	−3.195E + 00	−3.322E + 00
	worse	−3.181E + 00	−3.203E + 00	−3.084E + 00	−3.203E + 00	−3.081E + 00	−3.322E + 00	−2.658E + 00	−3.202E + 00	−3.106E + 00	−3.203E + 00
*F*_21_	min	−1.015E + 01	−1.015E + 01	−1.015E + 01	−1.015E + 01	−1.015E + 01	−1.015E + 01	−1.015E + 01	−1.015E + 01	−1.005E + 01	−1.015E + 01
	std	1.108E-06	2.252E + 00	1.682E + 00	3.664E + 00	1.857E + 00	1.485E-01	4.122E-03	2.322E + 00	1.116E + 00	2.383E + 00
	avg	−1.015E + 01	−3.991E + 00	−9.774E + 00	−6.279E + 00	−9.393E + 00	−1.012E + 01	−1.015E + 01	−4.259E + 00	−5.305E + 00	−8.633E + 00
	median	−1.015E + 01	−2.683E + 00	−1.015E + 01	−5.078E + 00	−1.015E + 01	−1.015E + 01	−1.015E + 01	−2.683E + 00	−5.055E + 00	−1.015E + 01
	worse	−1.015E + 01	−2.683E + 00	−2.630E + 00	−2.630E + 00	−5.055E + 00	−9.489E + 00	−1.014E + 01	−2.630E + 00	−5.054E + 00	−5.055E + 00
*F*_22_	min	−1.040E + 01	−6.063E + 00	−1.040E + 01	−1.040E + 01	−1.040E + 01	−1.040E + 01	−1.040E + 01	−1.040E + 01	−5.088E + 00	−1.040E + 01
	std	1.339E-06	1.159E + 00	2.361E + 00	3.786E + 00	1.763E-04	1.578E-11	1.070E-02	2.870E + 00	3.658E-04	2.661E + 00
	avg	−1.040E + 01	−3.649E + 00	−9.072E + 00	−6.365E + 00	−1.040E + 01	−1.040E + 01	−1.039E + 01	−4.957E + 00	−5.087E + 00	−8.285E + 00
	median	−1.040E + 01	−2.766E + 00	−1.040E + 01	−4.427E + 00	−1.040E + 01	−1.040E + 01	−1.039E + 01	−3.724E + 00	−5.087E + 00	−1.040E + 01
	worse	−1.040E + 01	−2.752E + 00	−5.087E + 00	−2.752E + 00	−1.040E + 01	−1.040E + 01	−1.036E + 01	−2.752E + 00	−5.086E + 00	−5.088E + 00
*F*_23_	min	−1.054E + 01	−1.054E + 01	−1.054E + 01	−1.054E + 01	−1.054E + 01	−1.054E + 01	−1.054E + 01	−1.054E + 01	−5.128E + 00	−1.054E + 01
	std	4.990E-06	3.282E + 00	2.755E + 00	3.930E + 00	1.951E-04	8.784E-12	9.657E-03	3.926E + 00	5.242E-04	1.199E + 00
	avg	−1.054E + 01	−5.216E + 00	−8.777E + 00	−6.753E + 00	−1.054E + 01	−1.054E + 01	−1.053E + 01	−5.418E + 00	−5.128E + 00	−1.027E + 01
	median	−1.054E + 01	−2.871E + 00	−1.053E + 01	−7.856E + 00	−1.054E + 01	−1.054E + 01	−1.053E + 01	−2.839E + 00	−5.128E + 00	−1.054E + 01
	worse	−1.054E + 01	−2.427E + 00	−2.807E + 00	−2.422E + 00	−1.054E + 01	−1.054E + 01	−1.050E + 01	−1.677E + 00	−5.126E + 00	−5.176E + 00

**Fig 6:**
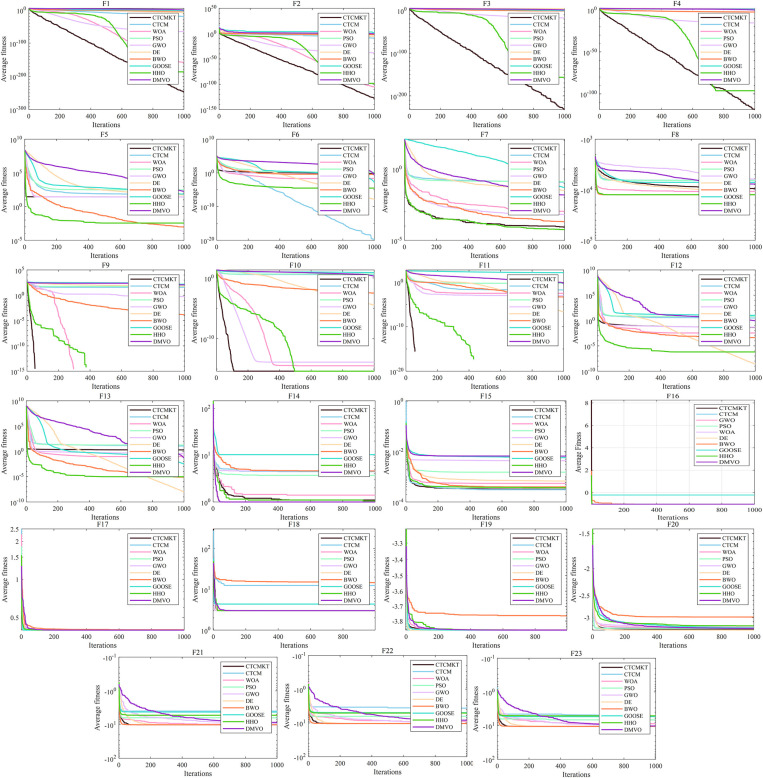
Convergence graphs of Benchmark functions.

Fig 6 presents the convergence curve graphs of the algorithms for 23 Benchmark functions. As shown in Fig 6, for functions *F*_1_ - *F*_4_ and *F*_7_, the CTCMKT algorithm exhibits the most rapid convergence speed and does not suffer from premature convergence. This implies that the algorithm has a robust capacity for searching and evading local optimal solutions. For functions *F*_5_ and *F*_6_, the initial iteration speed of the CTCMKT algorithm is higher than that of other algorithms, and it also ranks high in terms of convergence accuracy, which also means that the CTCMKT has better search capabilities. For the convergence curves of functions *F*_12_ and *F*_13_, the CTCMKT algorithm has relatively good accuracy and the fastest initial convergence speed compared to other algorithms. For *F*_9_ to *F*_11_, the CTCMKT algorithm not only converges the fastest but also has the best optimal value, which shows that the combined strategy of Kent chaotic mapping and *t*-distribution mutation can greatly improve the search ability and accuracy of the algorithm. In general, compared with other algorithms, CTCMKT has the best performance in global optimization of unimodal functions and multimodal functions. For function *F*_14_, the performance of the CTCMKT algorithm is second only to the DE when considering both accuracy and convergence speed. For functions *F*_15_-*F*_23_, the CTCMKT algorithm has extremely fast convergence speed and extremely high accuracy compared with other algorithms, which shows that the CTCMKT algorithm has a very good effect in optimizing fixed-dimensional multi-modal functions and has high global search capabilities and accuracy.

**Fig 7:**
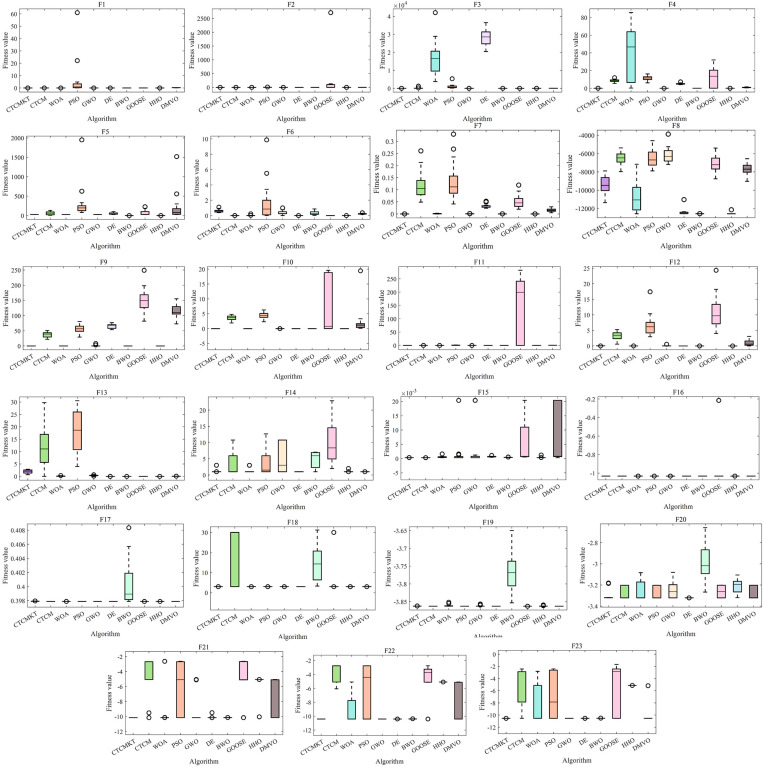
ANOVA tests for Benchmark functions.

The ANOVA test graphs for Benchmark functions are displayed in Fig 7. As shown in Fig 7 that the CTCMKT algorithm has the minimum standard deviation and high accuracy for function *F*_1_ to *F*_5_ and *F*_7_, which indicates that the CTCMKT possesses a strong ability to search and escape from the local optimal solution. For *F*_6_ and *F*_8_, the stability of CTCMKT has a high ranking. For functions *F*_9_ to *F*_12_, it can be seen from the Fig 7 that CTCMKT has the best stability and accuracy compared with other algorithms. For function *F*_13_, the standard deviation and optimal value of the CTCMKT algorithm are greatly improved compared with CTCM. For unimodal and multimodal functions, CTCMKT has smaller standard deviations and optimal values compared with several algorithms, which indicates that the Kent chaotic mapping combined with *t*-distribution mutation can improve the global search capability and stability of the algorithm. For functions *F*_15_-*F*_23_, the GWO, DE, BWO, HHO and CTCMKT algorithms all have relatively small standard deviations and optimal values in most test functions, and CTCMKT own smaller standard deviation and optimal value than CTCM. For multi-modal functions of fixed dimensions, the joint strategy to improve CTCM has a good effect, which can enhance the global optimization ability and stability of the algorithm and avoid premature convergence into the local optimal solution.

Table 10 shows the results of the Wilcoxon rank sum test. It can be seen from the data in the table that basically all p values are less than 0.05. For functions greater than 0.05, the comparative algorithm has found the optimal solution and the standard deviation is 0. Therefore, it can be shown that there are significant differences between CTCMKT and other functions.

**Table 10:** The Wilcoxon results for Benchmark functions.

*F*	CTCM	WOA	PSO	GWO	DE	BWO	GOOSE	HHO	DMVO
*F*_1_	6.796E-08	6.796E-08	6.796E-08	6.796E-08	6.796E-08	6.796E-08	6.796E-08	6.796E-08	6.796E-08
*F*_2_	6.796E-08	6.796E-08	6.796E-08	6.796E-08	6.796E-08	6.796E-08	6.796E-08	6.796E-08	6.796E-08
*F*_3_	6.796E-08	6.796E-08	6.796E-08	6.796E-08	6.796E-08	6.796E-08	6.796E-08	6.796E-08	6.796E-08
*F*_4_	6.796E-08	6.796E-08	6.796E-08	6.796E-08	6.796E-08	6.796E-08	6.796E-08	6.796E-08	6.796E-08
*F*_5_	5.250E-01	3.104E-01	6.796E-08	6.168E-01	6.674E-06	6.796E-08	7.577E-06	6.796E-08	1.201E-06
*F*_6_	6.796E-08	6.796E-08	4.094E-01	2.139E-03	6.796E-08	1.349E-03	6.796E-08	6.796E-08	6.796E-08
*F*_7_	6.796E-08	4.539E-07	6.796E-08	7.898E-08	6.796E-08	7.579E-04	6.796E-08	7.205E-02	6.796E-08
*F*_8_	9.173E-08	4.703E-03	6.796E-08	6.796E-08	9.173E-08	6.796E-08	2.563E-07	6.796E-08	2.690E-06
*F*_9_	8.007E-09	1.000E + 00	8.007E-09	8.773E-04	8.007E-09	8.007E-09	8.007E-09	1.000E + 00	8.007E-09
*F*_10_	8.007E-09	2.172E-05	8.007E-09	3.299E-09	8.007E-09	8.007E-09	8.007E-09	1.000E + 00	8.007E-09
*F*_11_	8.007E-09	3.421E-01	8.007E-09	1.626E-01	8.007E-09	8.007E-09	8.007E-09	1.000E + 00	8.007E-09
*F*_12_	6.796E-08	7.898E-08	6.796E-08	7.579E-04	6.796E-08	6.796E-08	6.796E-08	6.796E-08	1.610E-04
*F*_13_	1.610E-04	6.796E-08	6.796E-08	6.796E-08	6.796E-08	6.796E-08	6.796E-08	6.796E-08	6.796E-08
*F*_14_	3.560E-02	9.045E-03	9.674E-01	8.585E-02	8.007E-09	1.918E-07	9.160E-08	1.104E-05	1.065E-07
*F*_15_	6.776E-08	7.577E-06	3.150E-02	7.113E-03	6.796E-08	1.657E-07	6.796E-08	5.428E-01	9.127E-07
*F*_16_	8.007E-09	6.796E-08	1.127E-08	6.796E-08	8.007E-09	2.356E-06	1.227E-03	6.786E-08	7.948E-07
*F*_17_	8.007E-09	6.796E-08	8.007E-09	1.065E-07	8.007E-09	9.748E-06	6.796E-08	6.786E-08	6.796E-08
*F*_18_	1.052E-01	2.222E-04	2.946E-08	6.011E-02	4.352E-08	6.796E-08	1.376E-06	6.786E-08	6.674E-06
*F*_19_	8.007E-09	2.073E-02	8.007E-09	1.014E-03	8.007E-09	6.796E-08	6.796E-08	3.369E-01	6.796E-08
*F*_20_	2.011E-01	6.168E-01	8.041E-02	6.359E-01	1.958E-08	1.065E-07	4.249E-01	1.807E-05	8.585E-02
*F*_21_	6.484E-07	4.951E-08	5.909E-01	4.951E-08	1.161E-05	4.951E-08	3.603E-06	4.951E-08	4.951E-08
*F*_22_	4.458E-08	1.302E-05	5.927E-01	9.277E-07	2.074E-08	4.951E-08	1.089E-03	4.951E-08	5.940E-01
*F*_23_	6.724E-03	1.480E-05	1.000E + 00	1.066E-03	2.118E-08	6.031E-08	1.061E-01	6.031E-08	1.061E-01

**Fig 8:**
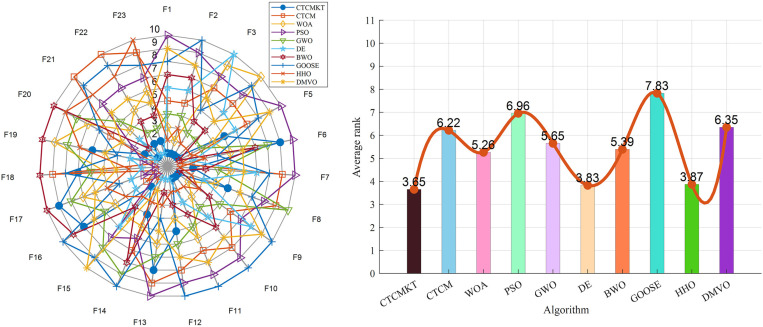
(a) Radar chart of these algorithms (23 Benchmark functions), (b) The sorting diagram of these algorithms (23 Benchmark functions).

The radar chart and sorting diagram of the 23 Benchmark functions are shown in Fig 8(a)–(b). From the radar chart, we can draw the following conclusions: CTCMKT ranked first in most functions, but ranked lower in *F*_6_ and *F*_16_ to *F*_19_. From the sorting diagram, we can see that CTCMKT ranked 3.65 overall, which is a significant improvement over the unimproved CTCM algorithm, and it also ranked first compared to other algorithms. This shows that the CTCMKT algorithm has a huge advantage in the 23 benchmark function tests, has better global optimization capabilities and stability, and can jump out of the local optimal solution and prevent premature convergence.

### CTCMKT for solving project optimization

For the purpose of checking the usability and practicality of the CTCMKT algorithm, it is applied to solve engineering optimization problems such as those related to compression spring projects, and welded beam design projects.

### Compression spring project

**Fig 9:**
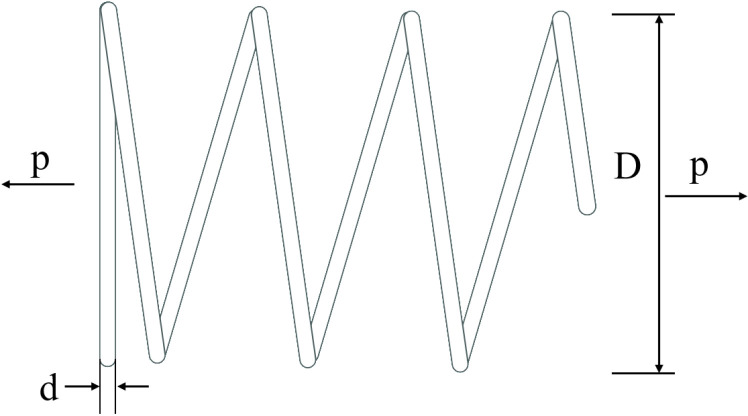
Compression spring project.

The objective in compression spring design is to reduce its mass *f*(x) to the minimum while adhering to specific constraints. These consist of four inequality constraints: minimum deflection, shear stress, oscillation frequency, and outer diameter limitation. There are also three design variables: the average diameter of the spring coil *D*(*x*_2_), the diameter of the spring wire *d*(*x*_1_), and the effective number of spring coils *N*(*x*_3_), as illustrated in Fig 9.

Minimize:


$$f(x)=(x_{3}+2)x_{2}{x_{1}}^{2}$$ (16)

subject to:


$$h_{1}(x)=1-\frac{{x_{2}}^{3}x_{3}}{71758{x_{1}}^{4}}\leq 0$$ (17)

$$h_{2}(x)=\frac{4{x_{2}}^{2}-x_{1}x_{2}}{12566(x_{2}{x_{1}}^{3}-{x_{1}}^{4})}+\frac{1}{5108{x_{1}}^{2}}-1\leq 0$$ (18)

$$h_{3}(x)=1-\frac{140.45x_{1}}{{x_{2}}^{2}x_{3}}\leq 0$$ (19)

$$h_{4}(x)=\frac{x_{2}+x_{1}}{1.5}-1\leq 0$$ (20)

with bounds:

$0.05\leq x_{1}\leq 2$,$0.25\leq x_{2}\leq 1.3$,$2\leq x_{3}\leq 15.$ (21)

**Fig 10:**
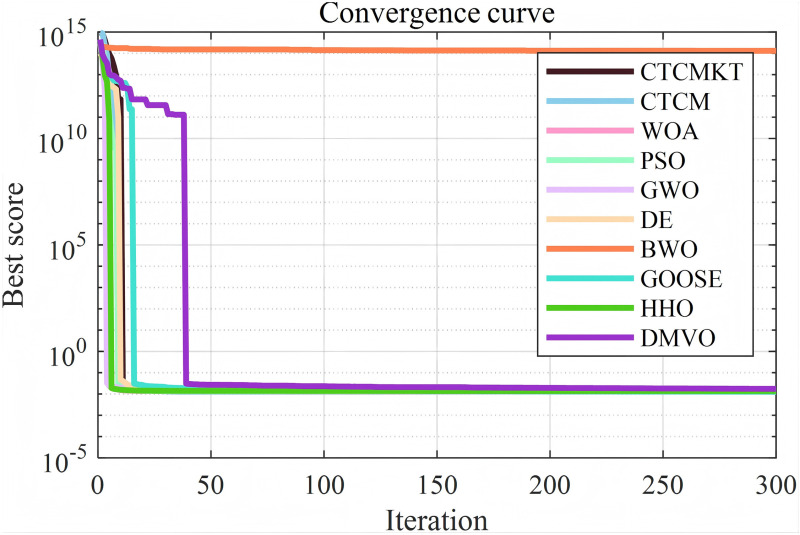
Convergence graphs of compression spring project.

**Fig 11:**
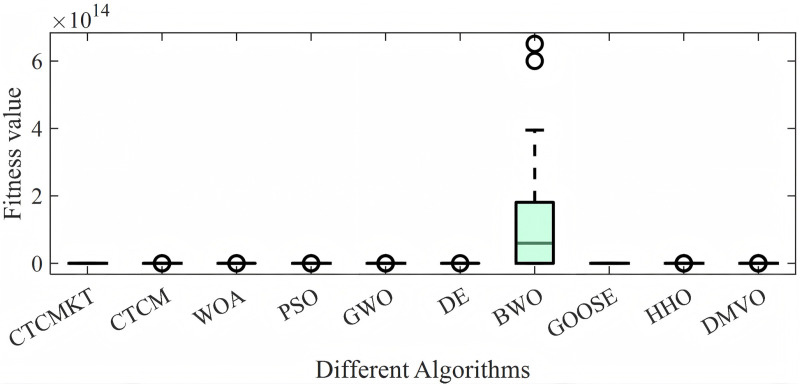
ANOVA tests of compression spring project.

For the compression spring project, the algorithm parameters are set as follows: the population size is 40, and the maximum number of iterations is 300. All algorithms are run 30 times to obtain the optimal value, average value, standard deviation and other statistical results as shown in Table 11. The convergence curve and ANOVA test graph are shown in Fig 10 and Fig 11, respectively. It can be seen from Fig 10 except for the BWO and DMVO, other algorithms have similar convergence speeds and optimal values. Similarly, in terms of the stability of the optimal solution for the compression spring, except for the BWO algorithm, the stability of other algorithms is good. As can be seen from Table 11, the CTCMKT algorithm has a smaller standard deviation than the CTCM algorithm. Except for the BWO algorithm, the other algorithms have almost the same optimal value. In general, the CTCMKT algorithm has strong global optimization capabilities and high stability to solve practical engineering application problems.

**Table 11:** Experimental statistical results for compression spring project.

Algorithm	CTCMKT	CTCM	WOA	PSO	GWO	DE	BWO	GOOSE	HHO	DMVO
min	0.0127	0.0130	0.0127	0.0127	0.0127	0.0127	0.0164	0.0127	0.0127	0.0128
worst	0.0132	0.0138	0.0161	0.0172	0.0134	0.0137	6.50E + 14	0.0134	0.0178	0.0193
std	0.0001	0.0002	0.0009	0.0011	0.0002	0.0002	1.76E + 14	0.0002	0.0015	0.0016
avg	0.0128	0.0133	0.0137	0.0134	0.0128	0.0130	1.30E + 14	0.0129	0.0139	0.0174
median	0.0127	0.0133	0.0135	0.0130	0.0127	0.0130	5.96E + 13	0.0128	0.0132	0.0180

### Welded beam design

**Fig 12:**
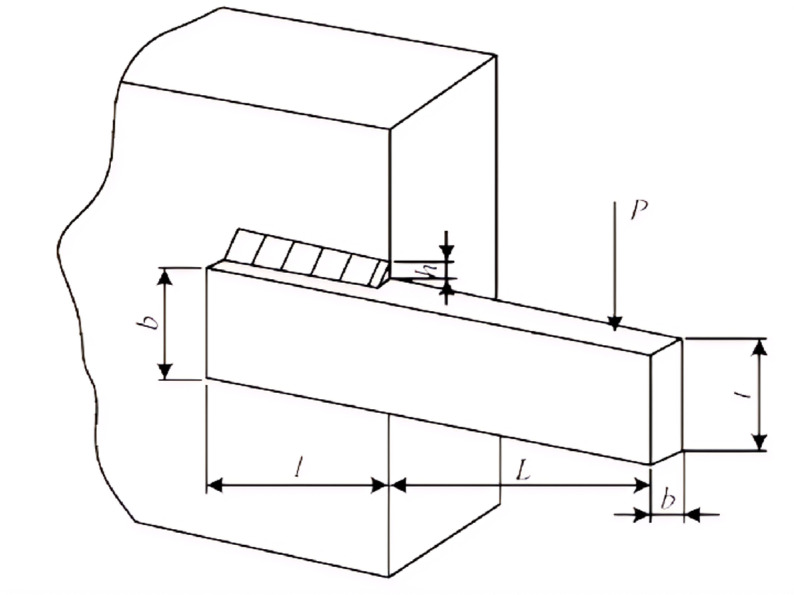
Welded beam design project.

The aim of welded beam design is to minimize its cost *f*(x) subject to specific constraints. There are seven inequality constraints involved, such as shear stress (*τ*), beam bending stress (*σ*), bar buckling load (*P*_C_), beam end deflection (*δ*), etc. The four design variables are: *h*(*x*_1_), *l*(*x*_2_), *t*(*x*_3_) and *b*(*x*_4_), as shown in Fig 12. The mathematical model is described in the form of Eq. (22).

Minimize:


$$f(x)=1.1047x_{1}^{2}x_{2}+0.04811x_{3}x_{4}(14.0+x_{2})$$ (22)

Subject to


$$y_{1}(x)=\tau (x)-\tau_{\max}\leq 0$$ (23)

$$y_{2}(x)=\sigma (x)-\sigma_{\max}\leq 0$$ (24)

$$y_{3}(x)=x_{1}-x_{4}\leq 0$$ (25)

$$y_{4}(x)=0.104x_{1}^{2}+0.04811x_{3}x_{4}(14.0+x_{2})-5.0\leq 0$$ (26)

$$y_{5}(x)=0.125-x_{1}\leq 0$$ (27)

$$\begin{array}{c} y_{6}(x)=\delta (x)-\delta_{\max}\leq 0 \end{array}$$ (28)

$$y_{7}(x)=P-P_{c}(x)\leq 0$$ (29)

Boundary constraints and related parameters.


$$\begin{array}{c} \tau (x)=\sqrt{{\left(\tau^{\prime }\right)}^{2}+2\tau^{\prime }\tau^{\prime \prime }\frac{x_{2}}{2R}+{\left(\tau^{\prime \prime }\right)}^{2}},\tau^{\prime }=\frac{P}{\sqrt{2}x_{1}x_{2}},\tau^{\prime \prime }=\frac{MR}{J}\\ M=P\left(L+\frac{x_{2}}{2}\right),R=\sqrt{\frac{x_{2}^{2}}{4}+{\left(\frac{x_{1}+x_{3}}{2}\right)}^{2}},\\ J=2\left[\sqrt{2}x_{1}x_{2}\left\{\frac{x_{2}^{2}}{12}+{\left(\frac{x^{1}+x^{3}}{2}\right)}^{2}\right\}\right] \end{array}$$ (30)

$$\sigma (x)=\frac{6PL}{x_{3}^{2}x_{4}},\delta (x)=\frac{4PL^{3}}{Ex_{3}^{3}x_{4}}$$ (31)

$$\begin{array}{c} P_{c}(x)=\frac{4.013E\sqrt{\frac{x_{3}^{2}x_{4}^{6}}{36}}}{L^{2}}\left(1-\frac{x_{3}}{2L}\sqrt{\frac{E}{4G}}\right),\\ P=6000lb,L=14in,E=30e6psi,G=12e6psi,\\ \tau_{\max}=13600psi,\sigma_{\max}=30000psi,\delta_{\max}=0.25in \end{array}$$ (32)

$$0.1\leq x_{1}\leq 2.0,0.1\leq x_{2}\leq 10.0,0.1\leq x_{3}\leq 10.0,0.1\leq x_{4}\leq 2.0$$ (33)

**Fig 13:**
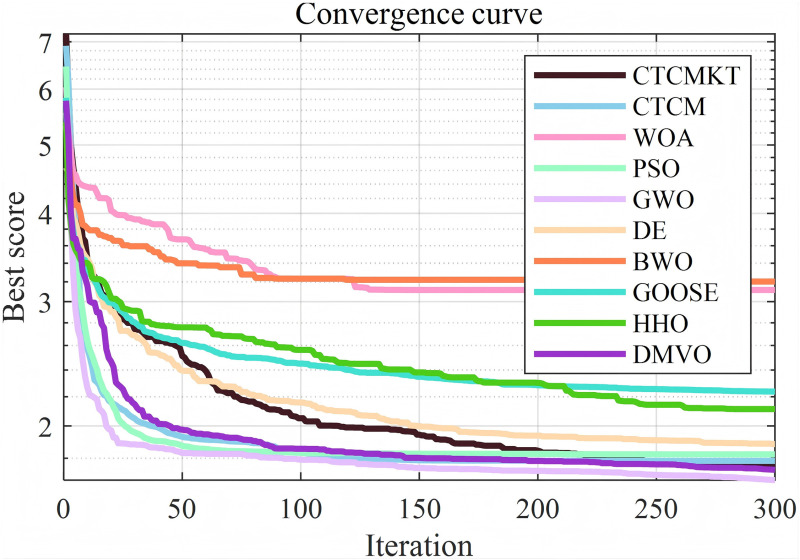
Convergence graphs of welded beam design project.

**Fig 14:**
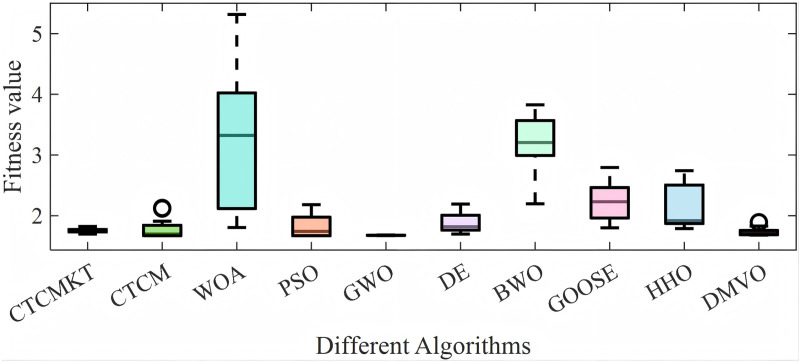
ANOVA tests of welded beam design project.

The statistical results of welded beam project are as shown in Table 12. The convergence curve and ANOVA test graph are shown in Fig 13 and Fig 14, respectively. As can be seen from Fig 13, all algorithms behave good convergence speed, and CTCMKT, PSO, GWO, HHO, DMVO show good global optimization capabilities. In terms of ANOVA test, CTCMKT showed very good stability, only slightly worse than GWO. As shown in Table 12, the standard deviation of CTCMKT is 0.0317 which is much better than 0.1571 of CTCM algorithm. In terms of average value, the average value of CTCMKT algorithm is 1.7547, which is also better than CTCM. In general, the CTCMKT algorithm is only weaker than the GWO algorithm in solving the welding beam design problem. Therefore, it can be shown that the Kent chaotic mapping combined with *t*-variation distribution can well improve the global optimization ability and stability of the CTCM algorithm and avoid falling into the local optimal solution.

**Table 12:** Statistical results of welded beam project.

Algorithm	CTCMKT	CTCM	WOA	PSO	GWO	DE	BWO	GOOSE	HHO	DMVO
min	1.6972	1.6702	1.8047	1.6702	1.6721	1.6972	2.1955	1.8002	1.7868	1.6790
worst	1.8223	2.1269	5.3167	2.1817	1.6813	2.1925	3.8276	2.7947	2.7424	1.8919
std	0.0317	0.1571	1.1172	0.1880	0.0025	0.1482	0.4336	0.2958	0.3455	0.0660
avg	1.7547	1.7844	3.1161	1.8231	1.6760	1.8851	3.2031	2.2372	2.1130	1.7331
median	1.7598	1.6970	3.3228	1.7420	1.6763	1.8193	3.2055	2.2301	1.9173	1.7048

In summary, the CTCMKT algorithm has obvious advantages in testing single-peak and multi-peak functions, but it is slightly insufficient in optimizing multi-peak functions of fixed dimensions. Other improvement strategies can be used to enhance its global optimization ability in the future. In solving engineering problems, the CTCMKT algorithm performs well. In the future, the CTCMKT algorithm can be used in path planning, material composition optimization, etc. to verify the actual application ability of the algorithm.

## Conclusion and future research

In conclusion, based on the joint strategy of Kent chaotic map and *t*-distribution mutation, this paper proposed an enhanced CTCM algorithm for function optimization and engineering problem solving. The CTCMKT algorithm improves the CTCM algorithm through a joint strategy, effectively avoids falling into the local optimal solution, and improves the stability and convergence speed of the algorithm. Compared with other algorithms, the CTCMKT algorithm has better standard deviation and optimal value in function testing, representing strong stability and global optimization ability. From the convergence curve, the CTCMKT algorithm has a faster convergence speed and accuracy. The CTCMKT algorithm has obvious advantages in testing unimodal and multimodal functions, but is slightly insufficient in optimizing multimodal functions of fixed dimensions. Its global optimization capability can be enhanced through other improvement strategies or by mixing other algorithms. For engineering optimization problems, the CTCMKT algorithm greatly improves the global optimization capability and robustness of the CTCM algorithm. From the experimental results, the CTCMKT algorithm effectively improves the convergence speed and accuracy of the algorithm, and can be used to solve practical engineering application. However, the ability of this optimization algorithm to solve other applications, such as three-dimensional path planning and material composition optimization, remains to be verified.

In future work, other intelligent optimization algorithms will be mixed to improve the algorithm’s global optimization ability to avoid premature convergence and algorithm stability. The enhanced CTCM algorithm can be used in engineering problems such as tracked mountain vehicle path planning, drone three-dimensional path planning, material composition optimization and ship track planning. The purpose of optimization is to find the path with the least time and the shortest distance to reduce time cost and fuel consumption. However, no single optimization algorithm can be all-encompassing, and much research remains to be done.
